# Significance of neutrophil microparticles in ischaemia‐reperfusion: Pro‐inflammatory effectors of endothelial senescence and vascular dysfunction

**DOI:** 10.1111/jcmm.15289

**Published:** 2020-06-10

**Authors:** Ali El Habhab, Raed Altamimy, Malak Abbas, Mohamad Kassem, Lamia Amoura, Abdul Wahid Qureshi, Hanine El Itawi, Guillaume Kreutter, Sonia Khemais‐Benkhiat, Fatiha Zobairi, Valérie B. Schini‐Kerth, Laurence Kessler, Florence Toti

**Affiliations:** ^1^ INSERM (French National Institute of Health and Medical Research) UMR 1260 Regenerative Nanomedicine (RNM) University of Strasbourg Illkirch-Graffenstaden France; ^2^ UMR CNRS 7213 Laboratory of Biophotonics and Pharmacology Faculty of Pharmacy University of Strasbourg Illkirch-Graffenstaden France; ^3^ Faculty of Pharmacy University of Strasbourg Illkirch-Graffenstaden France; ^4^ Department of Diabetes and Nutrition Endocrinology University Hospital of Strasbourg Strasbourg France; ^5^ Faculty of Medicine Federation of Translational Medicine (FMTS) Strasbourg France

**Keywords:** endothelial senescence, inflammation, ischaemia‐reperfusion, microparticles, transplantation, vascular dysfunction

## Abstract

Endothelial senescence is an emerging cause of vascular dysfunction. Because microparticles are effectors of endothelial inflammation and vascular injury after ischaemia‐reperfusion, we examined leucocyte‐derived microparticles of spleen origin as possible contributors. Microparticles were generated from primary rat splenocytes by either lipopolysaccharide or phorbol‐myristate‐acetate/calcium ionophore, under conditions mimicking innate and adaptive immune responses. Incubation of primary porcine coronary endothelial cells with either type of microparticles, but not with those from unstimulated splenocytes, leads to a similar threefold raise in senescence‐associated β‐galactosidase activity within 48 hours, indicating accelerated senescence, to endothelial oxidative stress, and a fivefold and threefold increase in p21 and p16 senescence markers after 24 hours. After 12‐hour incubation, the endothelial‐dependent relaxation of coronary artery rings was reduced by 50%, at distinct optimal microparticle concentration. In vitro, microparticles were pro‐thrombotic by up‐regulating the local angiotensin system, by prompting tissue factor activity and a secondary generation of pro‐coagulant endothelial microparticles. They initiated an early pro‐inflammatory response by inducing phosphorylation of NF‐κB, MAP kinases and Akt after 1 hour, and up‐regulated VCAM‐1 and ICAM‐1 at 24 hours. Accordingly, VCAM‐1 and COX‐2 were also up‐regulated in the coronary artery endothelium and eNOS down‐regulated. Lipopolysaccharide specifically favoured the shedding of neutrophil‐ and monocyte‐derived microparticles. A 80% immuno‐depletion of neutrophil microparticles reduced endothelial senescence by 55%, indicating a key role. Altogether, data suggest that microparticles from activated splenocytes prompt early pro‐inflammatory, pro‐coagulant and pro‐senescent responses in endothelial cells through redox‐sensitive pathways. The control of neutrophil shedding could preserve the endothelium at site of ischaemia‐reperfusion–driven inflammation and delay its dysfunction.

## 1 INTRODUCTION

Endothelial damage is a prime sensor of ischaemia‐reperfusion and a potential inducer of pro‐coagulant and pro‐inflammatory responses via leucocyte and platelet recruitment associated with oxidative stress, all characterizing ischaemia‐reperfusion injury (IRI). IRI is a major cause of graft damage characterized by oxidative stress and inflammation. Upon restoration of blood flow, accumulation of reactive oxygen species (ROS) and the release of cytokines prompt the endothelial up‐regulation of adhesion molecules, and the consecutive monocyte and neutrophil recruitment and extravasation into the post‐ischaemic tissues.^1^ Chemokines also favour neutrophil and monocyte recruitment in the early stages of reperfusion of transplanted organs,^2^ thereby amplifying IRI‐induced inflammation.^3^ Consequently, graft damage would favour immediate or acute rejection peri‐transplantation, as typified in pancreatic islet transplantation which is associated with a particular form of IR termed instant blood‐mediated inflammatory reaction (IBMIR).^4^, ^5^ The noxious interaction between damaged endothelial cells (ECs) and leucocytes demonstrated ex vivo, and in transplantation^6^ or thrombosis animal models,^7^ suggests that IRI‐driven initial graft damages would favour immediate or acute peri‐transplantation rejection through vascular dysfunction.

Circulating endothelial cells and endothelial‐derived microparticles (EMPs) are a signature of endothelial damage^8^ post‐IRI in humans. Elevated plasma levels of EMPs were associated with cardiac rejection and the duration of cold‐ischaemia.^9^ Conversely, in patients with renal failure, post‐transplantation EMP levels decrease, indicating endothelial recovery.^10^ Microparticles (MPs), also referred to as microvesicles, are vesicles shed from the plasma membrane of activated cells released in body fluids.^11^ MPs harbour cell membrane proteins and contain active lipids, proteins and mRNA, making them pro‐inflammatory and pro‐coagulant cellular effectors. They also identify the cell lineage they were stemmed from.^12^, ^13^ A common feature of MPs is their pro‐coagulant properties relying on (a) the exposure of phosphatidylserine that catalyses the assembly of blood coagulation complexes and (b) the eventual presence of active tissue factor (TF), the cellular initiator of blood coagulation inducible at endothelial and leucocyte surfaces.

Aside from the well‐established deleterious release of pro‐coagulant MPs during myocardial infarction, IR could also favour MP‐driven graft dysfunction through multiple cell lineages. Pro‐coagulant MPs were identified in the plasma of patients with islet graft rejection, possibly in association with IR,^14^, ^15^ while elevated TF^+^‐MPs characterized myocardial rejection^16^ and worsened outcome in stem cell transplantation.^17^

Pro‐inflammatory MPs from platelets, lymphocytes and monocytes would favour leucocyte recruitment at the inflamed endothelium surface known to expose adhesion molecules, like ICAM‐1, VCAM‐1 and E‐selectin, thereby accelerating IRI.^18^, ^19^, ^20^ Interestingly, a recent report describes a cardio‐splenic axis that augments infarct size during post‐ischaemic reperfusion via leucocyte activation and the recruitment of spleen neutrophils at site of the ischaemic heart,^21^ eventually favouring local endothelial dysfunction.

The impact of immune cells during IR remains unexplored with respect to endothelial senescence and vascular dysfunction. Endothelial senescence shifts the endothelium to a pro‐coagulant and pro‐inflammatory status with major TF activity, endothelial dysfunction and pro‐senescent EMP shedding,^22^, ^23^ and favours the development of a senescence‐associated secretory phenotype (SASP), mainly consisting of cytokines and metalloproteases.^24^

To decipher the inflammation‐driven endothelial damages during IRI, we used the rat spleen as a source of immune cell lineages and assessed the effects of MPs generated from splenocytes (SMPs), under conditions mimicking post‐ischaemic stress on endothelial accelerated senescence and dependent vascular function by in vitro and ex‐vivo SMP‐mediated crosstalk models.

## 2 MATERIALS AND METHODS

### 2.1 Animals

Male Wistar rats (300 g, Janvier Labs) were maintained on a standard 12‐hour light/dark cycle. Experiments conformed to the Guide of Care and the Use of Laboratory Animals published by the NIH (No. 85‐23, revised 1996) were authorized by the French Ministry of Higher Education and Research and by the local ethic committee (authorization 03799.01) and were done in the registered animal yard (number E‐67‐218‐26) of the Faculty of Pharmacy.

### 2.2 Rat splenocyte isolation and culture

After sacrifice, spleens were withdrawn, weighed and washed in phosphate‐buffered saline (PBS) before homogenization under sterile conditions and further filtered through 100‐µm sterile cell strainers. Following 300 *g* centrifugation for 5 minutes, the cell pellet was re‐suspended in a 5‐mL ammonium‐chloride‐potassium erythrocyte lysis buffer for 5 minutes, centrifuged (300 *g*, 5 minutes), washed in PBS and re‐suspended in FBS‐supplemented complete RPMI‐1640 medium and seeded into T75 culture flask at 5.10^6^ cells/mL.

### 2.3 Generation, isolation, quantification and characterization of splenocyte‐derived MPs

Splenocytes were stimulated by 5 µg/mL of lipopolysaccharide (LPS) (0127: B8; Sigma) or a combination of phorbol 12‐myristate 13‐acetate (PMA) (25 ng/mL; Enzo) and A12387 calcium ionophore (1 µmol/L; Sigma) at 37°C for 24 hours. In some experiments, splenocytes were washed after stimulation and incubated with an antibody cocktail for 20 minutes on ice. Leucocytes were characterized by flow cytometry (FACS 543 FORTESSA^TM^), according to a standardized in‐home protocol using FITC, BB700, PE, PE‐Cy7, APC, BV421, BV650 or BUV737 labelled monoclonal antibodies against rat CD3, CD4, CD45R, CD11b/c, CD8, CD25, CD11b (BD Pharmingen) and CD14 (Biotechne). Data were analysed by FlowJo (Treestar^®^).

MPs from LPS (SMP_LPS_), PMA/I (SMP_PMA/I_) or untreated splenocyte (SMP_CTL_) supernatants were washed, concentrated by differential centrifugation and re‐suspended in Hanks Balanced Salt Solution (HBSS) (supporting information). SMP measurement was performed by pro‐thrombinase assay after capture onto Annexin V‐coated micro‐wells using a microplate spectrophotometer and their concentration referred to as phosphatidylserine (PhtdSer) equivalent, by reference to a standard curve made with synthetic vesicles of known amounts of PhtdSer. In this assay, PhtdSer. is the rate‐limiting factor of prothrombin conversion into thrombin.^25^ The size distribution analysis of SMPs was performed by Tunable Resistive Pulse Sensing technology (TRPS) using the Izon qNano system and Izon control suite 3.3 software (Izon Science Ltd). Characterization of the SMP cell origin was performed by capture onto biotinylated antibodies directed against leucocyte CDs before quantification by pro‐thrombinase assay (supporting information). The SMP concentration was obtained by subtracting the OD values measured using istotype control biotinylated IgGs.

### 2.4 Isolation and treatment of Coronary Artery Primary Endothelial Cells

Pig hearts were collected from the local slaughterhouse (COPVIAL, Holtzheim, France) and ECs freshly isolated from the left circumflex coronary arteries (supporting information).

Young passage 1 ECs (P1ECs) were seeded at 75%‐80% confluence and incubated with 1‐30 nmol/L PhtdSer eq. SMP_CTL_, SMP_LPS_ or SMP_PMA/I_ for 30 minutes to 48 hours in complete MCDB medium at 37°C. Endothelial replicative senescence was induced by serial passaging (P3ECs) and premature senescence by a 48‐hour incubation with 100 µmol/L H_2_O_2_ of P1ECs^22^ and evidenced by the presence of senescence‐associated β‐galactosidase (SA‐β‐gal) activity.^26^ In different sets of experiments and before addition of SMPs, P1ECs were treated by inhibitors of PI3 kinase (PI3Ki, LY294002), p38 MAPK (p38i, SB203580) or ERK ½ (ERKi, PD98059) for 1 hour; an NADPH oxidase inhibitor (VAS‐2870), a cyclooxygenase inhibitor (indomethacin) or combined mitochondrial inhibitors of the respiratory chain (MKR) (myxothiazol + KCN+rotenone) for 30 minutes; or inhibitors of TLR‐dependent and TLR‐independent inflammatory signalling (N‐(2‐Morpholinylethyl)‐2‐(3‐nitrobenzoylamido)‐benzimidazole, Interleukin‐1 Receptor‐Associated‐Kinase‐1/4 Inhibitor (IRAKi)) or (5Z)‐7‐Oxozeaenol, *Curvularia sp* (TAK‐1i), a potent ATP‐competitive irreversible inhibitor of ERK2, TAK‐1 (MMK7) and MEK1 inhibitor, for 1 hour.

### 2.5 Measurement of apoptosis and senescence‐associated β‐galactosidase activity

Apoptosis and SA‐β‐gal were measured by flow cytometry using propidium iodide and AnnexinV (PI/AV) double labelling, or the C12FDG fluorogenic cell‐permeable substrate. SA‐β‐gal was revealed on the EC monolayer by microscopy using the X‐gal chromogenic substrate (supporting information).

### 2.6 Kinetics of SMP transfer to target endothelial cells

SMP (30‐60 nmol/L) was stained by 2 μmol/L of the red fluorescent PKH26 lipid probe (Sigma). PKH26‐stained SMPs were washed twice by centrifugation (14 000 *g*, 60 minutes) in HBSS at 4°C before incubation with P1ECs during 6‐48 hours. Capture of PKH26‐stained SMPs by target ECs was assessed after three washings by fluorescent microscopy (Leica FW 4000) and quantified by flow cytometry. The efficacy of the SMP capture was expressed as the percentage of red fluorescent ECs (Guava Easy Cyte Plus System, Millipore).

### 2.7 Western blot

Experiments were performed as described in supporting information.

### 2.8 Measurement of cellular and mitochondrial oxidative stress

P1ECs were incubated with SMP_LPS_ or SMP_PMA/I_ or by 100 µmol/L H_2_O_2_ as a positive control of oxidative stress‐induced senescence. P1ECs were treated by 2.5 μmol/L dihyroethidium, a redox‐sensitive red fluorescent dye, for 30 minutes or by 5 μmol/L MitoSOX^TM^ Red, a mitochondrial superoxide indicator, for 10 minutes at 37°C and assessed by flow cytometry after three washings. ROS were expressed as a fold increase by comparison with untreated P1ECs (P1 = 100%). Auto‐fluorescence gains were set at the first logarithmic decade (supporting information).

### 2.9 Tissue factor activity

Endothelial TF activity was measured by Tenase assay in 96‐well plates (5.10^4^ ECs/well). After washing by HBSS, purified human Factor X (150 nmol/L, Hyphen Biomed), Factor VIIa (5 nmol/L, NovoSeven) and 1 mmol/L CaCl_2_ were incubated for 30 minutes. Conversion of Factor X into Xa by TF was revealed by the cleavage of a specific chromogenic substrate (CS11, 0.1 mmol/L, Hyphen Biomed). Variations in absorbance were recorded using a microplate spectrophotometric reader in kinetic mode set at 405 nm (Molecular Device). Data are expressed as fM of active TF per 5.10^4^ living ECs by reference to a standard curve established with known amounts of highly purified lipidated recombinant human TF (ADF Biomedical).

### 2.10 Vascular reactivity

Vascular reactivity of coronary artery rings was assessed as previously described.^27^ Briefly, the coronary artery was cut into 2‐3 mm length rings, incubated 12 hours under sterile conditions with SMPs and suspended in organ baths containing oxygenated (95% O_2_, 5% CO_2_) Krebs bicarbonate solution (NaCl 119 mmol/L, KCl 4.7 mmol/L, KH_2_PO_4_ 1.18 mmol/L, MgSO_4_ 1.18 mmol/L, CaCl_2_ 1.2 mmol/L, NaHCO_3_ 25 mmol/L and D‐glucose 11 mmol/L, pH 7.4, at 37°C). After equilibration, rings were pre‐contracted with the thromboxane mimetic U46619 (1‐60 nmol/L) before construction of concentration‐response curves to bradykinin. Relaxations are expressed as a percentage of the contraction to U46619.

### 2.11 Immunofluorescence microscopy of coronary arteries

Pig coronary arteries were incubated with SMPs for 24 hours before OCT inclusion and immunostaining (supporting information).

### 2.12 Selective depletion of splenocyte‐derived microparticles

Immuno‐magnetic depletion of SMP of specific cell origins was achieved using biotinylated antibodies insolubilized on streptavidin‐coated beads (50 μL beads‐300 μL of SMPs) and directed against either rat neutrophil CD11b, monocytes/macrophages CD11b/c or endothelial CD31 (DynabeadsM‐280, Thermo Fisher). Control isotype IgG‐coated beads served as control. Immuno‐depletion was achieved by the addition of 1 μg of a chosen antibody‐coated bead suspension to the concentrated SMPs and the mixture further incubated for 30 minutes at 4°C, before a 2‐minute contact with the magnet. A double depletion step allowed 75%‐80% of specific SMP depletion by pro‐thrombinase capture assay.

### 2.13 Statistical analysis

Data, expressed as mean ± standard error mean (SEM), were analysed using GraphPad Prism5^®^. Statistical analysis was performed using one‐way ANOVA test followed by the Tukey's post hoc or Mann‐Whitney tests. A *P* < .05 was considered significant. All measurements were from at least three separate individuals.

## 3 RESULTS

### 3.1 Differential impact of LPS or PMA/I treatments on splenocyte cell subsets

**FIGURE 1:**
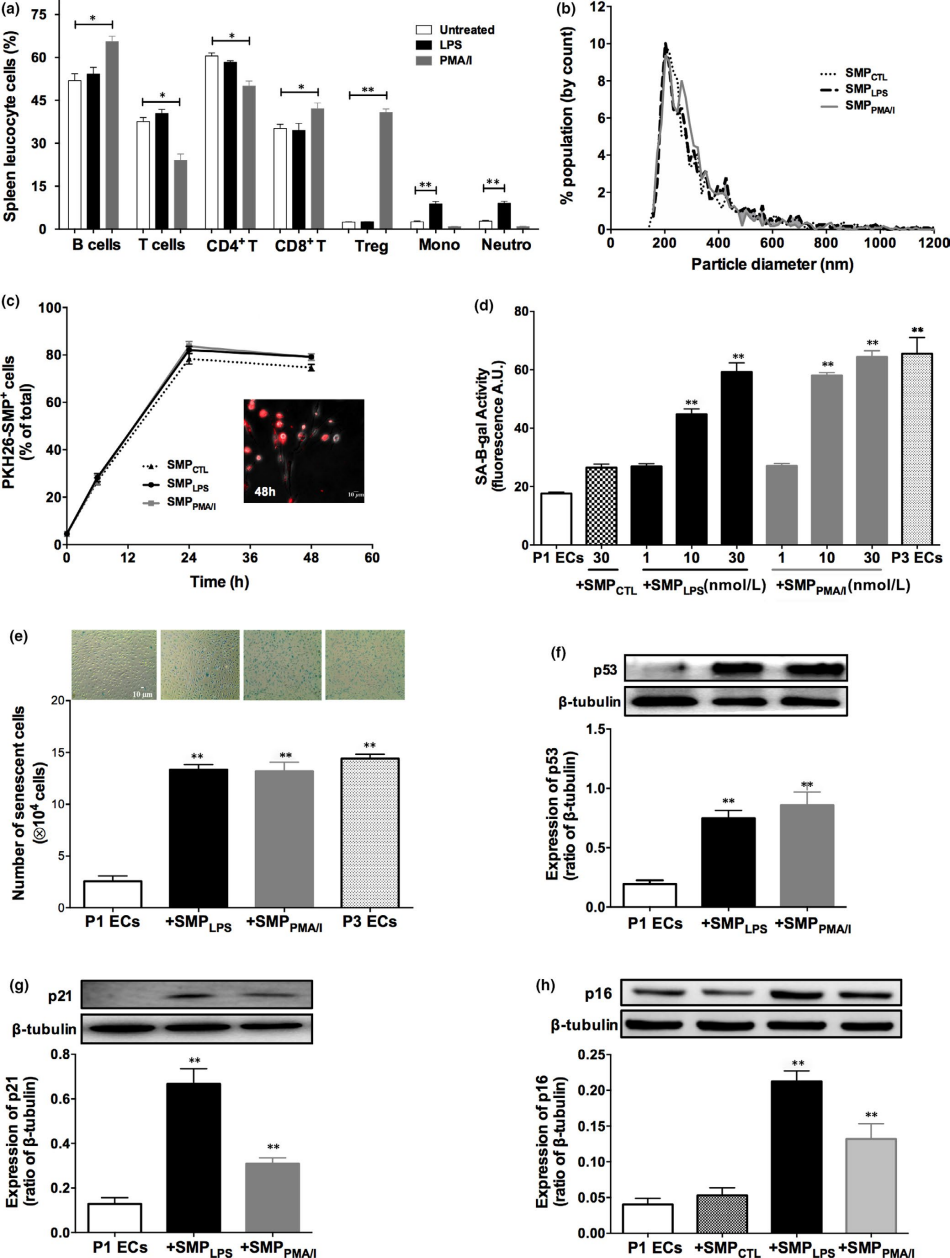
LPS or PMA/I have distinct effects on splenocyte lineages and released SMPs that induce premature senescence in target porcine P1ECs. A, Immune phenotyping of leukocyte subsets in the rat spleen. After 24‐h stimulation by either 5 μg/mL LPS or a combination of 25 ng/mL PMA and 1 μmol/L A23187 calcium ionophore, cultured splenocytes were washed and stained with fluorescent labelled antibodies and were analysed by flow cytometry. Histograms show the percentages of spleen leukocyte subsets in the indicated samples. B, qNano generated data of splenocytes microparticles (SMP_CTL_, SMP_LPS_ or SMP_PMA/I_). Plot depicts particle size diameter vs. percentage (%) of population. C, P1ECs were incubated with SMP_CTL_, SMP_LPS_ or SMP_PMA/I_ labelled with the red fluorescent lipid probe PKH26. Kinetics of SMP_CTL_, SMP_LPS_ or SMP_PMA/I_ integration by P1ECs measured by flow cytometry. Data expressed as the percentage of PKH26‐labelled SMP‐positive P1ECs. No fluorescence signal was measured in the absence of SMP incubation (not shown). The micrograph is representative of the fluorescent signal obtained after incubation for 48 h with PKH26+‐SMP_LPS_ in four different experiments performed likewise. D, SA‐β‐gal activity in P1ECs after 48‐h incubation with either splenocyte microparticles harvested in the supernatant of untreated splenocytes (SMP_CTL_, 30 nmol/L), different concentrations of SMP_LPS_ or SMP_PMA/I_ (1‐30 nmol/L). SA‐β‐gal activity was assessed using the C12FDG fluorogenic substrate by flow cytometry. E, P1ECs were incubated for 48 h with 10 nmol/L SMP_LPS_ or SMP_PMA/I_ before assessment of SA‐β‐gal activity using the X‐gal chromogenic substrate. F‐H, P1ECs were incubated for 24 h with 30 nmol/L SMP_LPS_ or SMP_PMA/I_ before analysis of the expression of p53 (F), p21 (G) and p16 (H) senescence markers by Western blot. Immunoblots (upper panel) and densitometry analysis of cumulative data (lower panel). Data are expressed as mean ± SEM of experiments performed at least on three different cell cultures. P1ECs: untreated P1 endothelial cells, SMP_CTL_: SMPs from unstimulated splenocytes; SMP_LPS_: SMPs from LPS‐stimulated splenocytes; SMP_PMA/I_: SMPs from PMA/I‐stimulated splenocytes, P3ECs: Senescent cells. *P < .05, **P < .01

The leukocyte cell phenotype pattern differed with the splenocyte treatment, indicating distinct triggering of adaptive and innate immunity by PMA/I and LPS. PMA/I mainly altered lymphocyte subsets with enhanced proportions of B cells, CD8^+^‐T lymphocytes and CD4^+^‐CD25^+^ T regulatory cells and decreased CD4^+^ T Lymphocytes. Conversely, LPS tripled the proportion of neutrophil and monocyte subsets (Figure 1A; Figure S1).

### 3.2 LPS or PMA/I induces the generation of splenocyte‐derived microparticles that interact with young endothelial cells

Compared to vehicle, a significant SMP release was measured by pro‐thrombinase assay after 24 hours, by, respectively, 2.5‐ and 3.5‐fold in response to LPS (SMP_LPS_) or PMA/I (SMP_PMA/I_), together with a twofold and threefold increase in splenocyte apoptosis (Figure S2A,B). In accordance with the above splenocyte phenotyping, the SMP cell origin varied with the inducer: LPS induced a significant 2.5‐fold raise in both CD11b^+^‐ and CD11b/c^+^‐SMPs indicating a major activation of the innate immune cells. By contrast, CD8b^+^‐, CD4^+^‐, CD161a^+^‐ and CD25^+^‐SMPs concentrations remained unchanged. Conversely, PMA/I induced a significant raise in CD8b^+^‐ (16‐fold), CD4^+^‐ (sevenfold) and CD25^+^‐SMPs (sevenfold), representative of lymphocyte activation with no modification in CD11b^+^‐ and CD11b/c^+^‐SMPs concentrations (Table S1). TRPS measurement showed no alteration in SMP size distribution by any of the inducers, with a size range of 170‐220 nm, peaking at a 208 nm median diameter (Figure 1B).

Interestingly, kinetics of PKH26‐labelled SMPs integration into target plasma membrane of ECs were similar for all SMPs, 28% of fluorescent P1ECs being stained after 6 hours, the proportion increasing thereafter to a 82% plateau at 24 hours, by flow cytometry (Figure 1C).

### 3.3 SMP_LPS_ or SMP_PMA/I_ are specific inducers of premature senescence in target P1ECs with no pro‐apoptotic effect

SMP_LPS_ or SMP_PMA/I_ but not SMP_CTL_ (*P* = .1002) markedly increased SA‐β‐gal activity measured by flow cytometry in target P1ECs after 48 hours. Significant elevations were observed in response to 10‐30 nmol/L of either SMP_LPS_ or SMP_PMA/I_, the higher concentration inducing a SA‐β‐gal activity similar to that observed in P3 senescent cells (Figure 1D). Simultaneously, the absence of apoptosis, even after 30 nmol/L SMPs treatment, suggested a specific pro‐senescent effect, as confirmed by PI/AV labelling and Western blot analysis of cleaved caspase‐3. Conversely, 100 µmol/L H_2_O_2_ prompted maximum EC apoptosis (33.67 ± 2.57% vs 9.34 ± 0.72% in untreated P1ECs, Figure S3A,B).

The pro‐senescent feature of SMP_LPS_ or SMP_PMA/I_ was also evidenced on EC monolayers by transmission microscopy, using the X‐Gal blue SA‐β‐gal substrate. A significant threefold increase in cells bearing SA‐β‐gal activity was induced after 48 hours, values reaching those observed in senescent P3 cells (Figure 1E). In addition, the expression of senescence markers increased as early as 24 hours after incubation with SMP_LPS_ or SMP_PMA/I_. A fourfold up‐regulation of p53, and a fivefold and threefold increase in down‐stream p21 and p16 proteins was respectively observed in P1ECs. The absence of SMP_CTL_‐driven up‐regulation of p16 (*P* = .7261) confirms the specific pro‐senescent properties of SMP_LPS_ or SMP_PMA/I_ (Figure 1F‐H).

The SMP‐driven pro‐senescent and specific effect was confirmed by the fact that concentrations of truly soluble LPS (0.5‐5 µg/mL) or PMA/I (2.5‐25 ng/mL/0.1‐1 µmol/L) remained unable to promote senescence. Furthermore, no soluble moiety from splenocyte supernatant could account for the induction of premature senescence, as incubation of P1ECs with SMP‐free conditioned medium did not lead to senescence (Figure S4A,B).

### 3.4 SMP_LPS_ or SMP_PMA/I_ induce oxidative stress in target P1ECs

**FIGURE 2:**
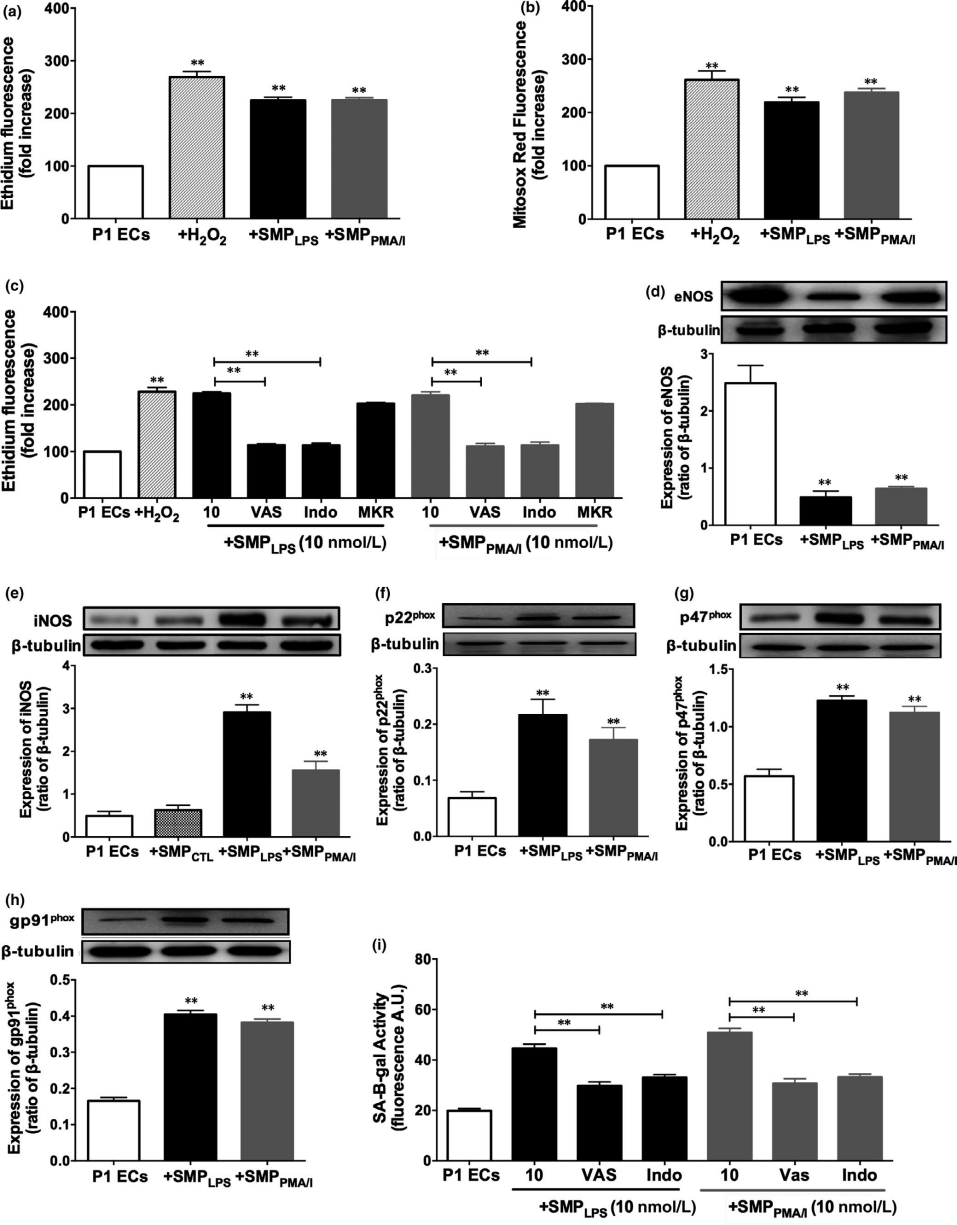
SMP_LPS_ or SMP_PMA/I_ promote early oxidative stress and the up‐regulation of pro‐oxidant enzymes in P1ECs. P1ECs were incubated for 6 h either with 100 μmol/L H_2_O_2_, 10 nmol/L SMP_LPS_ or SMP_PMA/I_ before the determination of oxidative stress using the redox‐sensitive probe DHE (A), of mitochondrial ROS using MitoSOX Red (B). C, P1ECs were incubated for 30 min with inhibitors of either NADPH oxidase (VAS), cyclooxygenase (Indo) or of the mitochondrial respiratory chain complex (MKR) before the addition of 10 nmol/L SMP_LPS_ or SMP_PMA/I_ for 6 h, and the subsequent measurement of oxidative stress. D‐H, P1ECs were incubated for 24 h with 30 nmol/L SMP_LPS_ or SMP_PMA/I_ before determination of the expression of eNOS (D), iNOS (E) and NADPH oxidase subunits p22phox (F), p47phox (G) and p91phox (H) by Western blot. Immunoblots (upper panel) and densitometry analysis of cumulative data (lower panel). I, P1ECs were incubated for 30 min with inhibitors of either NADPH oxidase (VAS, VAS‐2870, 5 μmol/L) or cyclooxygenase (Indo, indomethacin, 30 μmol/L) before the addition of 10 nmol/L SMP_LPS_ or SMP_PMA/I_ for 48 h, and the assessment of SA‐β‐gal activity by flow cytometry. Data are expressed as mean ± SEM of experiments performed at least on three different cell cultures. **P < .01

As ROS are strong inducers of senescence, we examined their variations in 10 nmol/L SMP_LPS_ or SMP_PMA/I_‐induced premature senescence. An early significant twofold ROS increase was induced after 6 hours in P1ECs cytoplasm and mitochondria (Figure 2A,B). By comparison, only a 2.7‐fold ROS enhancement was measured in H_2_O_2_‐induced apoptotic ECs (Figure 2A). These data suggest that, even at low concentration, SMPs promote oxidative stress that may be causative in the induction of senescence.

The source of ROS was further characterized using inhibitors of the major vascular sources of ROS including NADPH oxidase, cyclooxygenases and the mitochondrial respiration complex. VAS‐2870 and indomethacin blunted 50 ± 2.3% of either SMP_LPS_‐ or SMP_PMA/I_‐induced ROS after 6 hours, whereas the mitochondrial respiration complex inhibitors had only minor effects (Figure 2C). These findings suggest that NADPH oxidase and COXs contribute to the SMP_LPS_‐ or SMP_PMA/I_‐induced oxidative stress, as previously reported in response to pro‐senescent EMPs from coronary artery ECs.^22^

Because endothelial senescence is characterized by endothelial dysfunction and a reduced formation of nitric oxide (NO), we assessed SMP_LPS_ or SMP_PMA/I_ effects on eNOS expression. Western blot analysis indicated a respective 80.5 ± 3.1% and 75 ± 1.8% down‐regulation by SMP_LPS_ or SMP_PMA/I_, suggesting a blunted formation of NO and an increased iNOS expression whereas SMP_CTL_ had no effect (Figure 2D,E). In addition, p22^phox^, p47^phox^ and gp91^phox^ subunits of NADPH oxidase were up‐regulated, respectively, by a 2.5‐, 2.4‐ and 3.3‐fold range (Figure 2F‐H). Altogether, data confirm an unbalanced overproduction of ROS. Indeed, selective inhibitors of NADPH oxidase and COXs blunted either SMP_LPS_‐ or SMP_PMA/I_‐induced senescence measured by SA‐β‐gal activity (Figure 2I).

### 3.5 SMP_LPS_ or SMP_PMA/I_ promote a pro‐coagulant profile in target P1ECs by enhancing tissue factor activity and activate the local angiotensin system

**FIGURE 3:**
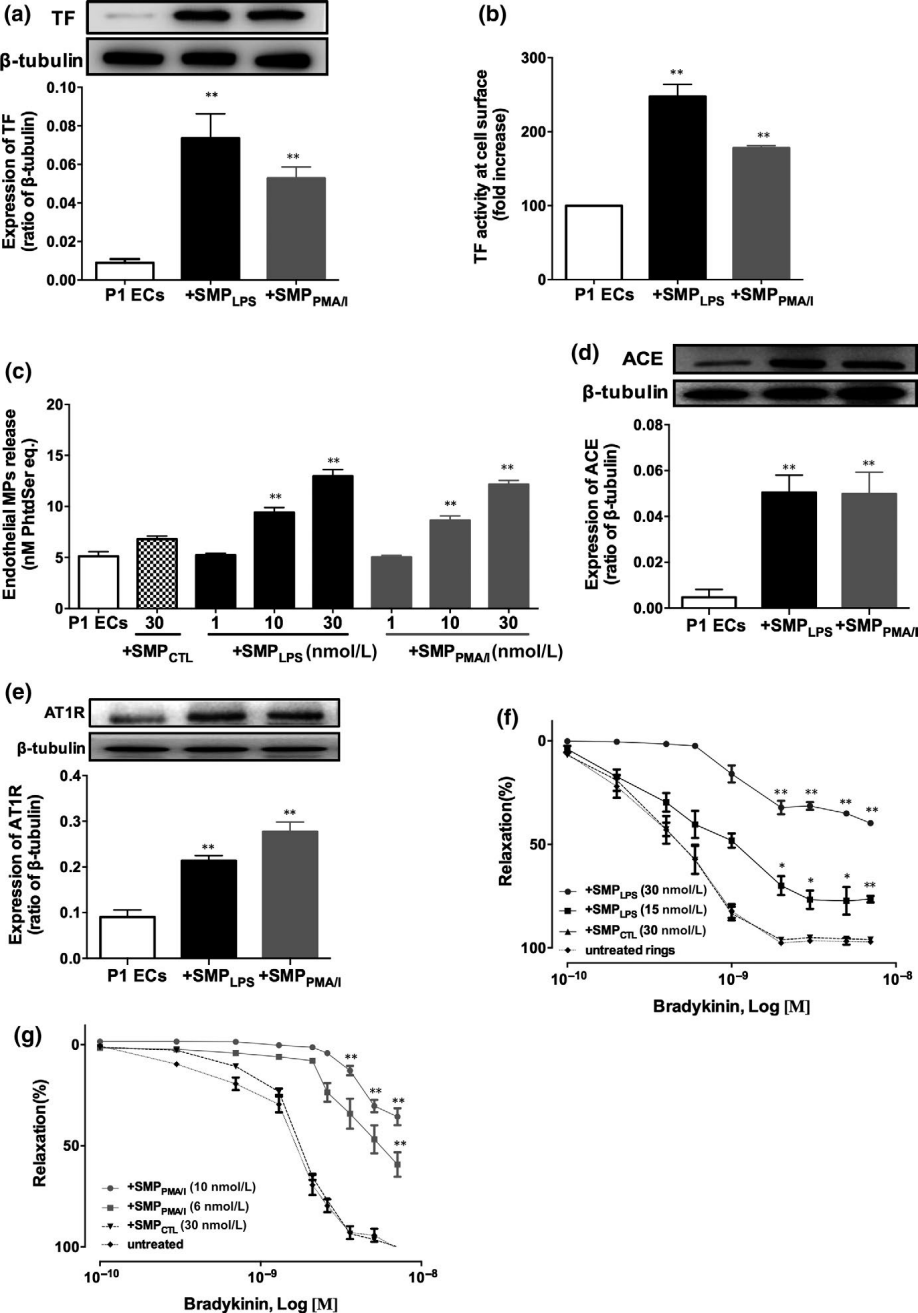
SMP_LPS_ or SMP_PMA/I_ induce early and sustained pro‐coagulant responses and promote the local angiotensin system in P1ECs, and blunt bradykinin‐induced endothelial relaxation in porcine coronary artery rings. A‐B, P1ECs were incubated with 10‐30 nmol/L SMP_LPS_ or SMP_PMA/I_ before determination of the expression of TF by Western blot after 24 h (A), and TF activity measured at cell surface after 6 h (B) by Tenase assay. Results expressed as fold increased Tenase activity in P1ECs: pMTF per 10^4^ cells. C, P1ECs were incubated for 24 h either with 30 nmol/L SMP_CTL_, different concentrations of SMP_LPS_ or SMP_PMA/I_ (1‐30 nmol/L) before measurement of endothelial MPs in the supernatant by pro‐thrombinase assay. D‐E, P1ECs were incubated for 24 h with 30 nmol/L SMP_LPS_ or SMP_PMA/I_ before determination of the expression of ACE (D) and AT1R (E) by Western blot. Immunoblots (upper panel) and densitometry analysis of cumulative data (lower panel). F‐G, Effect of SMPs on the vascular reactivity to bradykinin. Coronary artery rings were incubated for 12 h with different concentrations of SMP_LPS_ (F) or SMP_PMA/I_ (G) (1‐30 nmol/L) before construction of concentration‐response curves to bradykinin. Data are expressed as mean ± SEM of experiments performed at least on three different cell cultures. **P < .01

SMP_LPS_ or SMP_PMA/I_ shifted the endothelial phenotype to a pro‐coagulant status as shown by the respective ~sixfold and fourfold up‐regulation of TF expression (Figure 3A), and the 2.5‐fold and twofold enhanced TF activity (Figure 3B). Another SMP_LPS_‐ or SMP_PMA/I_‐driven pro‐coagulant response was the secondary shedding of EMPs detected by pro‐thrombinase assay, but not in response to SMP_CTL_ (SMP_LPS_: 12.7 nmol/L, SMP_PMA/I_: 12.17 nmol/L vs 6.8 nmol/L SMP_CTL_, *P* < .01, Figure 3C).

Because the local angiotensin system has been associated with endothelial senescence and vascular dysfunction, the expression of the angiotensin‐converting enzyme (ACE) and of the angiotensin receptor AT1 (AT1R) was assessed in SMP_LPS_‐ or SMP_PMA/I_‐treated P1ECs. ACE and AT1R were significantly up‐regulated, respectively, by fourfold and twofold (Figure 3D,E), suggesting a thrombogenic effect as reported.^22^

To confirm the SMP‐mediated endothelial dysfunction, SMP_LPS_ or SMP_PMA/I_ were incubated during 12 hours with porcine coronary artery rings before assessment of bradykinin endothelial‐induced relaxation. Both SMP_LPS_ and SMP_PMA/I_ blunted relaxation in a dose‐dependent manner, SMP_PMA/I_ being more efficient with a 50% inhibition measured in response to a 10 nmol/L concentration vs 30 nmol/L for SMP_LPS_ (Figure 3F,G). Of note, SMP_CTL_ had no effect, even at 30 nmol/L. The MP‐driven specific inhibition of relaxation was also confirmed by the fact that truly soluble 5 µg/mL of LPS or 25 ng/mL/1 µmol/L of PMA/I remained unable to alter bradykinin‐induced vaso‐relaxation (Figure S5A‐C). Furthermore, SMPs lysates had no effect, indicating the pivotal role of the SMP membrane proteolipid structure for endothelial targeting (data not shown).

### 3.6 SMP_LPS_ or SMP_PMA/I_ are endothelial pro‐inflammatory effectors

**FIGURE 4:**
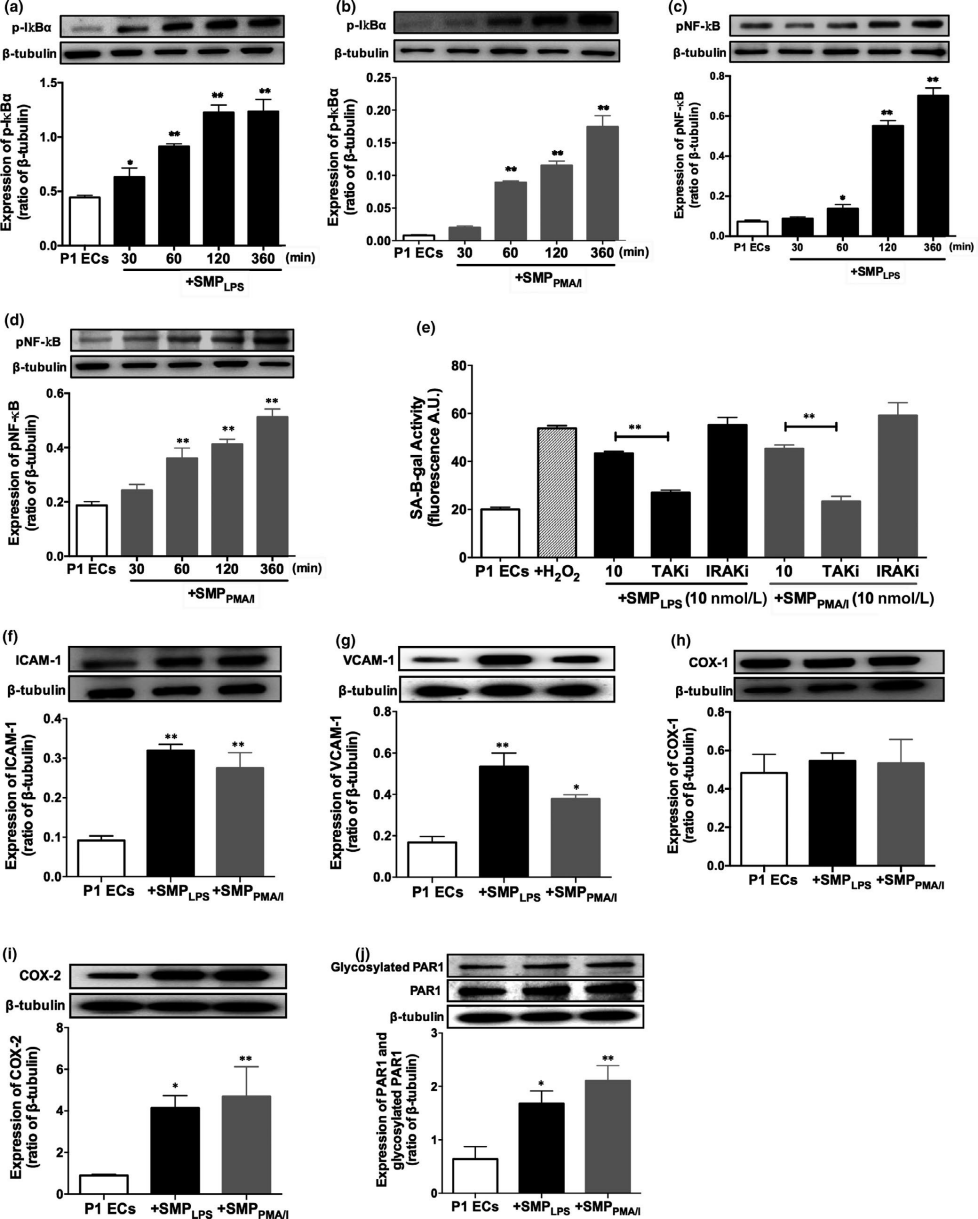
Pro‐senescent SMP_LPS_ or SMP_PMA/I_ trigger the NF‐κB pathway in P1ECs and up‐regulate inflammatory and adhesion proteins. P1ECs were incubated for 24 h with 30 nmol/L SMP_LPS_ or SMP_PMA/I_ before determination of the phosphorylation level of IκBα (A‐B) and NF‐κB (C‐D). Immunoblots (upper panel) and densitometry analysis of cumulative data (lower panel). E, P1ECs were incubated either with 100 μmol/L H_2_O_2_, and a selective inhibitor of TAK‐1 kinase (TAKi, (5z)‐7‐Oxozeanol, 10 μmol/L) or IRAK (IRAKi, 10 μmol/L) for 30 min prior to the addition of 10 nmol/L SMP_LPS_ or SMP_PMA/I_ for 48 h before the determination of SA‐β‐gal activity. F‐J, P1ECs were incubated for 24 h with 30 nmol/L SMP_LPS_ or SMP_PMA/I_ before determination of the expression of ICAM‐1 (F), VCAM‐1 (G), COX‐1 (H), COX‐2 (I), PAR‐1 and glycosylated PAR‐1 (J). Immunoblots (upper panel) and densitometry analysis of cumulative data (lower panel). Data are expressed as mean ± SEM of experiments performed at least on three different cell cultures. *P < .05, **P < .01

Because senescent cells secrete pro‐inflammatory cytokines and chemokines recruiting leucocytes and promoting endothelial inflammation, we assessed endothelial inflammatory markers in vitro and ex vivo. In P1ECs, both SMP_LPS_ and SMP_PMA/I_ induced an early and time‐dependent phosphorylation of IκBα (30 minutes up to 6 hours) and NF‐κB (1 hour up to 6 hours) (*P* < .01 vs P1ECs, Figure 4A‐D). The inflammatory potency of SMPs was totally abolished by 1 hour pre‐treatment with 10 µmol/L Tak‐1 inhibitor, whereas the IRAK inhibitor had no effect, thereby confirming the NF‐κB involvement, and indicating the contribution of ERK and JNK MAP kinases in the SMP‐induced senescence (Figure 4E).

After 24‐hour stimulation, SMP_LPS_ and SMP_PMA/I_ induced a significant and respective threefold and twofold up‐regulation of ICAM‐1 and VCAM‐1, and a fourfold increased COX‐2 expression, whereas COX‐1 remained unchanged (Figure 4F‐I). Interestingly, both SMPs up‐regulated the expression of PAR‐1 by 2.5‐ and threefold, respectively, and no modification of the proportion of its N‐terminal glycosylation could be observed, suggesting that the newly synthetized PAR‐1 would mainly remain inactivated (Figure 4J).

**FIGURE 5:**
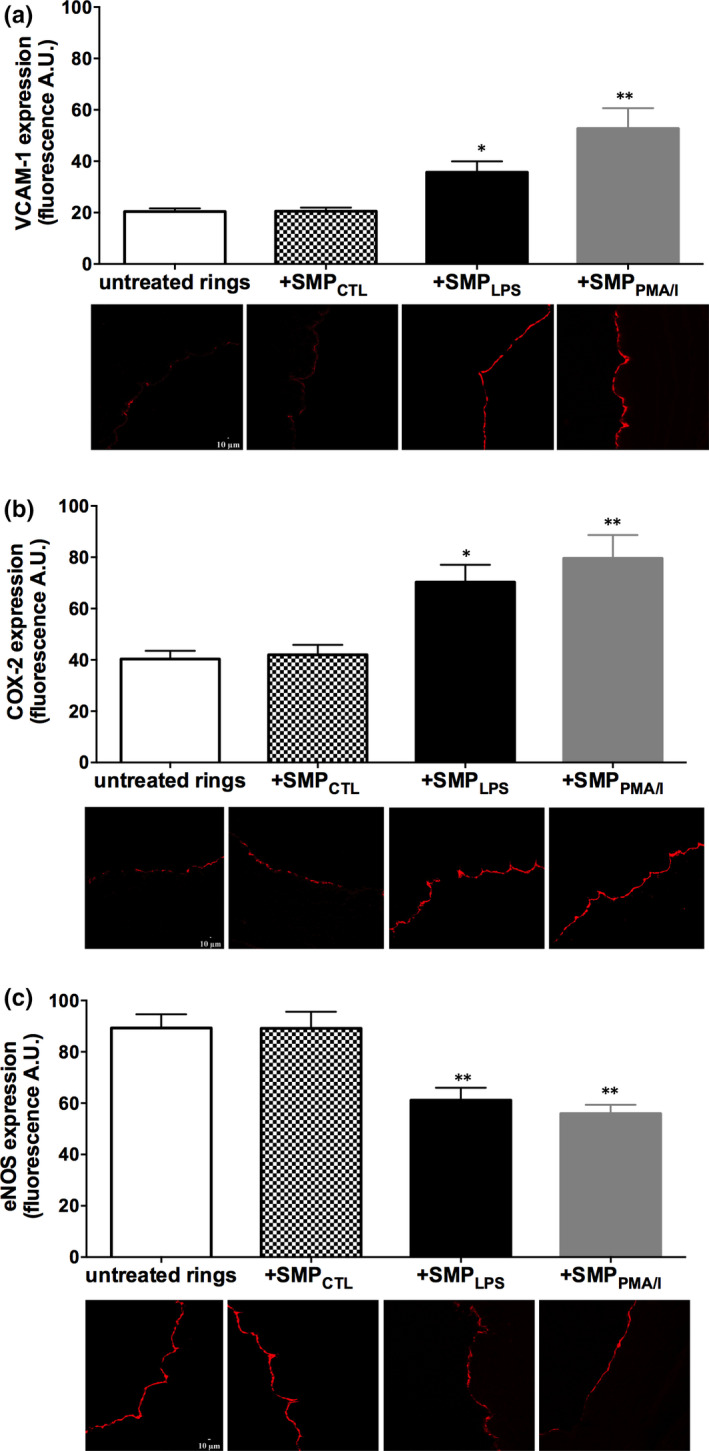
SMP_LPS_ or SMP_PMA/I_ induce the endothelial up‐regulation of VCAM‐1 and COX‐2 and the down‐regulation of eNOS in coronary artery rings. Coronary artery rings were incubated for 24 h with either 30 nmol/L SMP_CTL_, 10 nmol/L SMP_LPS_ or SMP_PMA/I_ before determination of the expression of VCAM‐1 (A), COX‐2 (B) and eNOS (C) by confocal fluorescence microscopy. Representative micrographs, with lumen facing left, showing fluorescent signal of different labelled antibodies at the endothelial monolayer surface. Data are expressed as mean ± SEM of experiments performed at least on three separate occasions. *P < .05, **P < .01

The potency of SMPs to act as vascular effectors was further confirmed in coronary artery tissues. SMP_LPS_ or SMP_PMA/I_ induced a significant endothelial expression of VCAM‐1 and COX‐2 in coronary artery rings and a 35% decrease in eNOS expression, by confocal fluorescence microscopy (Figure 5A‐C).

### 3.7 SMP_LPS_ or SMP_PMA/I_ prompt MAPKs and PI3 kinase pathways

**FIGURE 6:**
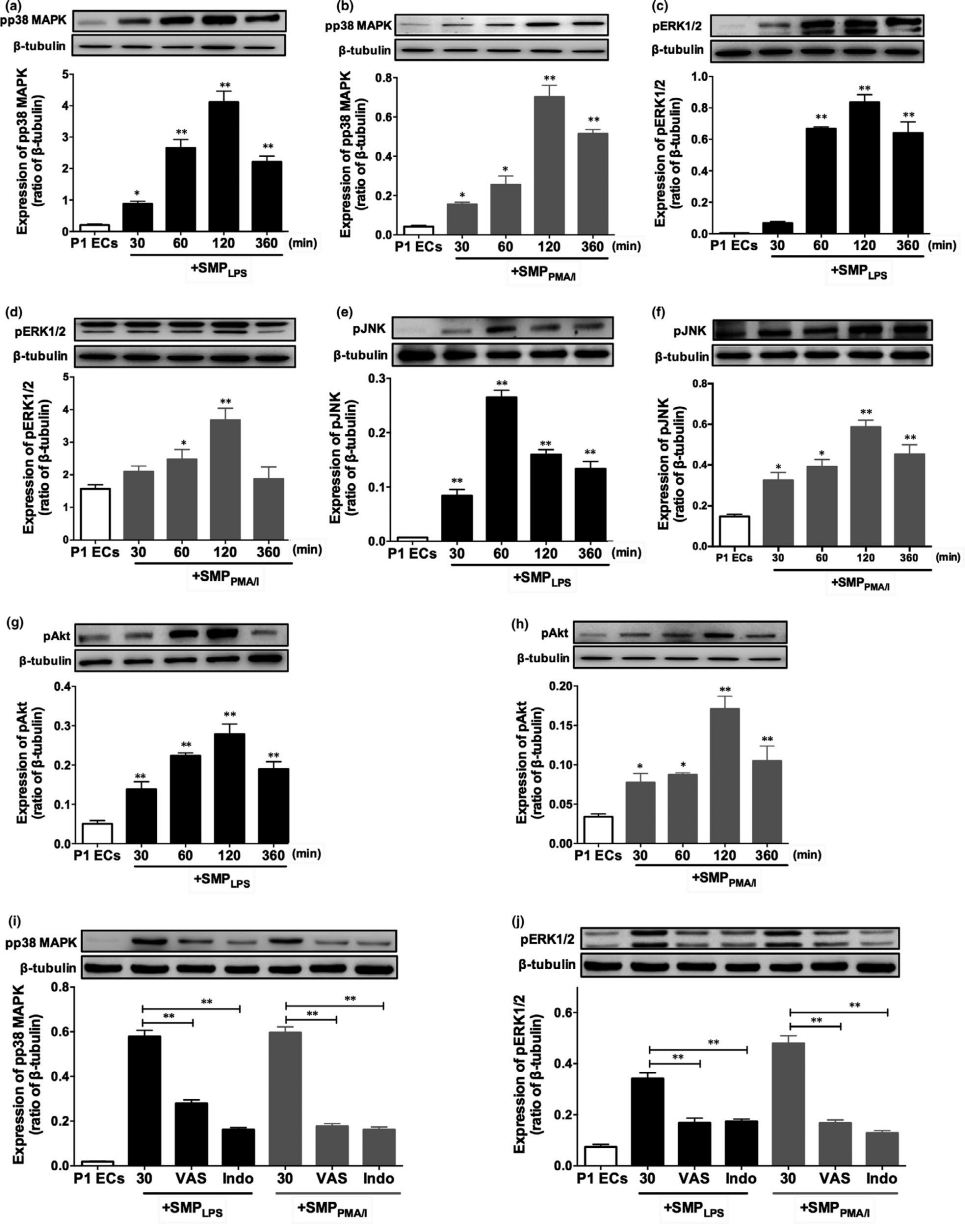
SMP_LPS_ or SMP_PMA/I_ trigger phosphorylation of p38 MAPK, ERK1/2, JNK and Akt in P1ECs. P1ECs were incubated for 24 h with 30 nmol/L SMP_LPS_ or SMP_PMA/I _before determination of the phosphorylation level of p38 MAPK (A‐B), ERK1/2 (C‐D), JNK (E‐F) and Akt (G‐H). P1ECs were incubated for 30 min with inhibitors of either NADPH oxidase (VAS, VAS‐2870, 5 μmol/L) or cyclooxygenase (Indo, indomethacin, 30 μmol/L) before the addition of 30 nmol/L SMP_LPS_ or SMP_PMA/I_ for 24 h and the determination of the phosphorylation level of p38 MAPK (I) and ERK1/2 (J). Immunoblots (upper panel) and densitometry analysis of cumulative data (lower panel). Data are expressed as mean ± SEM of experiments performed at least on three different cell cultures. *P < .05, **P < .01

As MAP kinases were involved in senescence signalling,^22^ we further assessed the role of redox‐sensitive kinases in SMP_LPS_‐ or SMP_PMA/I_‐induced premature endothelial senescence. In P1ECs, either SMP_LPS_ or SMP_PMA/I_ induced within 30 minutes the phosphorylation of p38 MAPK, ERK1/2, JNK and Akt, which persisted in a time‐dependent manner up to 2 hours incubation, descending after 6 hours (Figure 6A‐H). A 30 minutes pre‐treatment by selective inhibitors of NADPH oxidase and COXs confirmed redox‐sensitive MAP kinase mechanisms leading to senescence with a rapid limitation of p38 and ERK1/2 MAP kinase phosphorylation by at least 60%, 2 hours after incubation with SMP_LPS_ or SMP_PMA/I_ (Figure 6I,J).

**FIGURE 7:**
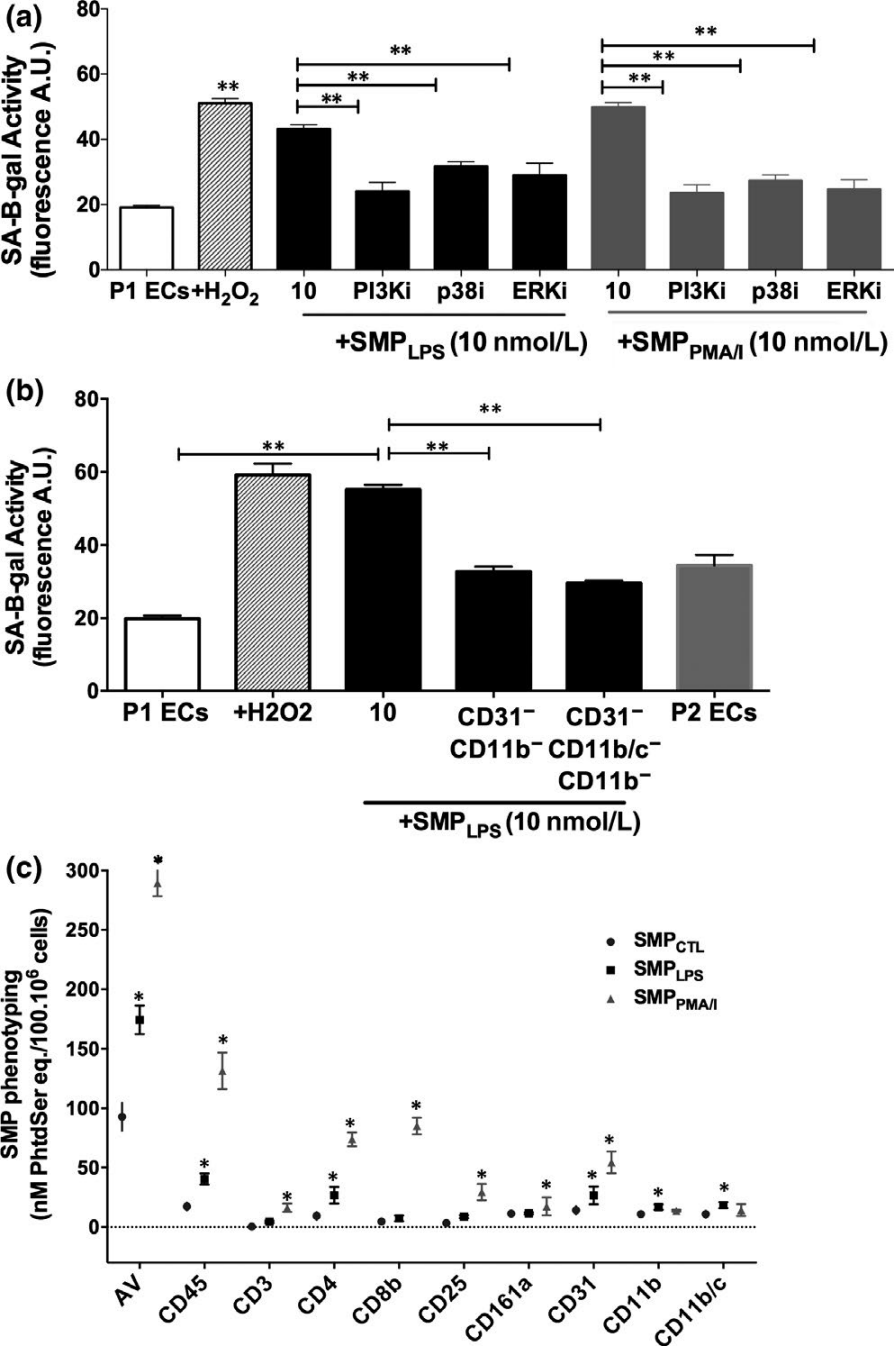
SMP_LPS_ induce endothelial senescence through MAP‐Kinase and PI3 kinase pathways, and via neutrophil‐derived MPs. A, P1ECs were incubated either with 100 μmol/L H_2_O_2_, or a selective inhibitor of PI3 kinase (PI3Ki, LY294002, 10 μmol/L), p38 MAPK (p38i, SB203580, 10 μmol/L) or ERK ½ (ERKi, PD98059, 10 μmol/L) for 1 h prior to the addition of 10 nmol/L SMP_LPS_ or SMP_PMA/I_ for 48 h before the determination of SA‐β‐gal activity. B, Effect of the removal of either CD31+/CD11b+‐MPs or CD31+/CD11b+/CD11b/c+‐ MPs on the endothelial pro‐senescent effect of SMP_LPS_. P1ECs were incubated with 10 nmol/L of SMP_LPS_ and with non‐depleted suspensions for 48 h before the determination of SA‐β‐gal activity. C, Characterization of the cell origin of SMP shed from splenocytes stimulated by LPS or PMA/calcium ionophore. After 24‐h stimulation, SMPs were measured in cell supernatants by pro‐thrombinase assay and expressed as nmol/L PhtdSer per 100.10^6^ cells. Characterization of the SMP cell origin was performed by capturing SMPs onto biotinylated antibodies directed against leucocyte CDs before quantification by pro‐thrombinase assay. Data are expressed as mean ± SEM of experiments performed at least on three different cell cultures. AV: Annexin V. *P < .05, **P < .01

In addition, a 30 minutes pre‐treatment by selective inhibitors of p38 MAPK, ERK1/2 and PI3 kinase significantly reduced SMP_LPS_‐ or SMP_PMA/I_‐induced senescence by 57 ± 3.3%, 54.7 ± 4.1% and 51 ± 3.6% respectively, as revealed by SA‐β‐gal activity measured after 48 hours (Figure 7A).

Altogether, data suggest that SMP_LPS_ or SMP_PMA/I_ induce premature ECs senescence through an early activation of the redox‐sensitive MAP‐Kinase and the PI3 kinase/Akt pathways.

### 3.8 Neutrophil‐ and monocyte‐derived SMPs contribute to early endothelial senescence

Because the SMP_LPS_ showed a specific profile with 65% elevation in neutrophil and monocyte cell origin compared to SMP_PMA/I_ (Table S1), we further characterized their specific contribution to SMP‐driven senescence. As we previously demonstrated that EMPs from senescent cells are pro‐senescent,^22^ endothelial CD31^+^‐SMP_LPS_ were depleted before specific removal of either neutrophil‐ or monocyte‐derived SMP_LPS_ (Table S2).

A 80% depletion in CD11b^+^‐SMPs and 75% in CD11b/c^+^‐SMPs reduced the SMP‐driven SA‐β‐gal activity by 55%‐60% after 48‐hour incubation, values of the treated cells returning close to those measured in pre‐senescent ECs at passage 2 (Figure 7B,C).

## 4 DISCUSSION

We report herein that MPs generated from LPS‐ or PMA/calcium ionophore‐treated splenocytes induce endothelial dysfunction and premature senescence via early NADPH oxidase‐dependent inflammatory responses. SMPs trigger NF‐κB and both MAPK and PI3 kinase/Akt pathways. Endothelial senescence was characterized by drastic eNOS down‐regulation, oxidative stress, up‐regulation of TF expression and enhanced activity, and the generation of secondary pro‐coagulant EMPs. The MP‐driven endothelial dysfunction was confirmed by the collapse of endothelial‐dependent vascular relaxation. Importantly, SMPs were strictly pro‐senescent with no pro‐apoptotic potential, indicating a specific effect.

### 4.1 Splenocytes as a valuable source of immune cell‐derived MPs

Because spleen was reported to exacerbate the inflammatory response during myocardial infarction and worsen the infarct size,^21^ we reasoned that SMPs would constitute a IRI‐driven inflammatory signature. Indeed, in rat models, an intraperitoneal sub‐septic LPS dose leads to transient cytokine secretion associated to early monocyte and neutrophil spleen recruitment within 3 hours.^28^ Consistently, in our primary splenocyte model, the SMP pattern is specific of the inducer, LPS favouring the release from neutrophils and monocytes, and PMA/I from lymphocytes. These yields are on line with the twofold to threefold raise in human monocyte‐ and T lymphocyte‐derived MPs reported in vitro after challenge by LPS and actinomycin D.^29^, ^30^, ^31^, ^32^

### 4.2 SMP_LPS _are early contributors to senescence

SMPs shared common endothelial pro‐senescent effects, with distinct impacts on key protein expression or activity. In ECs, SMP_LPS_ were more effective in the up‐regulation of senescent markers p16 and p21 and prompted higher pro‐coagulant and pro‐inflammatory typical responses like TF and VCAM‐1 up‐regulation, with an earlier IκBα activation, whereas MAP kinases, PI3 kinases and Akt were mostly phosphorylated at comparable time by both SMPs. Interestingly, SMP_LPS_ or SMP_PMA/I_ acted as differential vascular effectors in the blunting of endothelial‐dependent relaxation, SMP_PMA/I_ being more efficient. Immuno‐depletion assays confirmed that neutrophil‐ and monocyte‐derived SMP_LPS_ are key to premature endothelial senescence.

### 4.3 Key role of spleen neutrophils in the generation of pro‐senescent microparticles

Several previous reports, although not clearly deciphering the respective contribution of microparticles from exosomes, strongly suggest paracrine effects of circulating neutrophil microparticles (CNMPs) on various endothelial territories. In models of sepsis and inflammatory disorders including atherothrombosis, CNMPs disrupt the endothelial barrier and up‐regulate pro‐inflammatory mediators such as IL‐6, IL‐8 and ROS.^33^, ^34^, ^35^, ^36^ During inflammation, neutrophil migration from tissue compartments may partly account for the rapid shift of blood neutrophil count (10%‐25% in mice, 50%‐70% in human)^37^ and increased levels of CNMPs. Because the cell proportion of neutrophils still remains low in spleen or blood, the high potency of neutrophil‐derived microparticles is indirectly demonstrated by antibody abrogation that blunts the delivery of a microparticle signal to the endothelium.^38^, ^39^ Microparticle properties would however contribute to the initiation of inflammatory response or its resolution depending on the neutrophil activation kinetics, as demonstrated by RNA or protein detection.^36^, ^38^, ^39^, ^40^, ^41^ In our hands, a small proportion of spleen neutrophil (8%) contributed to the release of pro‐senescent microparticles in response to LPS after 24 hours, thereby underlying their high potency as tissue effectors of vascular dysfunction. Indeed, half SMP_LPS_‐driven endothelial senescence was abolished after their selective immuno‐depletion.

### 4.4 SMP_LPS_ or SMP_PMA/I_ as early pro‐inflammatory effectors of premature endothelial senescence triggered by innate immune cells

The pro‐senescent effect of both SMP_LPS_ and SMP_PMA/I_ was TAK kinase‐dependent with an early activation of NF‐κB only 1 hour after challenge, suggesting a SMP‐mediated pro‐inflammatory pathway. A 6‐hour SMP challenge only targeted 30% of ECs, indicating that the SMP‐mediated senescence is a feed‐forward process. Indeed, senescence decreased upon monocyte‐ or neutrophil‐derived SMP selective depletion, thereby strongly suggesting their early recruitment as a driving force to endothelial dysfunction.

Our observation of an early activation of p38 and JNK MAP kinases confirms the key role of pro‐inflammatory pathways and mediators in the SMP‐driven endothelial senescence. Accordingly, p38 MAPK activation was reported a redox‐sensitive contributor to senescence in HUVEC after serum starvation, using p38 silencing,^42^ and to the release of pro‐inflammatory EMPs from human aortic ECs in response to TNFα.^43^ Because NF‐κB signalling is involved in both endothelial SASPs and in the anti‐inflammatory control of senescence by caveolae initiating after 2 days and increasing thereafter in response to TNFα,^44^, ^45^ kinetics of SMP‐driven SASPs remain to be established beyond 48 hours to identify mechanisms of prolonged endothelial senescence. Altogether, monocyte and neutrophil MPs appear paracrine inductors of early endothelial inflammation, which in turn amplifies dysfunction and senescence, possibly via the secondary shedding of pro‐inflammatory EMPs already known as pro‐senescent effectors.^22^, ^23^, ^46^ In our model, as the depletion of monocyte‐ and neutrophil‐derived SMP_LPS_ maintains ECs at a pre‐senescent P2 stage, these MPs are likely initiators of the senescence responses. Nevertheless, the contribution of SMP_PMA,_ mainly of lymphocyte origin remains to be explored.

### 4.5 SMP_LPS_ or SMP_PMA/I_ as pro‐oxidant and pro‐coagulant endothelial effectors of coronary artery dysfunction

ROS are known mediators of both premature and replicative senescence in coronary and aortic ECs.^22^, ^23^, ^47^ Furthermore, EMPs released by premature or replicative senescent cells are autocrine inducers of superoxide anion via NADPH oxidase and cyclooxygenase, amplifying the endothelial dysfunction.^22^, ^23^ Similarly, using a pharmacological approach, we herein demonstrate that both SMP_LPS_ and SMP_PMA/I_ are pro‐senescent and promote a major early oxidative stress in ECs via NADPH oxidase and cyclooxygenase‐2. Our data do not confirm the ROS accumulation and enhanced NO production combined to unchanged eNOS activity previously reported in the Eahy.926 lineage treated by THP‐1–derived MPs.^48^ Discrepancies might rely on the initial stress (VP‐16–induced apoptosis) or on cell type.

Ageing is associated with progressive endothelial senescence and dysfunction and enhanced circulating MP levels,^49^, ^50^ while hampering graft survival.^51^, ^52^ Our ex vivo data showed that pro‐senescent SMPs blunt bradykinin‐induced relaxation of coronary arteries, indicating the collapse of endothelial NO‐mediated vaso‐protection owing to reduced eNOS expression and up‐regulated COX‐2, whereas SMP_CTL_ were ineffective. Taken together, our data confirm and extend previous reports indicating that the endothelium‐dependent vaso‐relaxation is a prime target of circulating pathological MPs isolated from patients with acute myocardial infarction.^53^

Other authors and our team have reported that angiotensin II triggers endothelial senescence and MP shedding^23^ through AT1R, enhanced ACE activity and redox‐sensitive pathways.^23^, ^47^ In accordance, we show herein that SMP‐induced premature endothelial senescence is characterized by up‐regulated AT1R and ACE. In the absence of AT1R mRNA quantification, the present data should however be balanced in view of the well‐known broad specificity of the antibodies against the AT1R.^54^ Nevertheless, we demonstrate an early enhancement in TF activity, in compliance with the reported TF‐associated endothelial dysfunction.^22^, ^55^ As the TF gene is early expressed in response to cytokines, it is tempting to anticipate that SMP‐driven endothelial senescence is linked to a rapid TF‐driven pro‐coagulant switch of the endothelium. Interestingly, no PAR‐1–mediated inflammatory response could be identified in spite of PAR‐1 up‐regulation after 24 hours. Possible explanations would be (a) an earlier burst of PAR‐1 glycosylation, (b) a counter‐regulation of the PAR‐1–induced chemokine and cytokine up‐regulation initiated by the suppressor of cytokine signalling 1 (SOCS‐1), as in human endometrial ECs.^56^, ^57^

Because VCAM‐1 and ICAM‐1 were up‐regulated in isolated ECs after 24 hours, or in the EC lining the rings, it can be anticipated that in the vessel, an acute generation of leukocyte MPs favours endothelial inflammation and consecutive senescence. Indeed, circulating leucocyte‐ and neutrophil‐derived MPs were demonstrated of prognosis value of primary and secondary worsen cardiac outcome.^58^, ^59^ Furthermore, circulating leucocyte MPs from septic rats prompt NF‐_K_B activation in the vessel wall and cardiac tissues, and are suitable candidates for pharmacological control.^60^ Interestingly, elevated plasma MP levels of endothelial and neutrophil cell origin characterize the severity of the coagulopathy and vascular damage in human sepsis, recently associated with NETosis and circulating neutrophil extracellular traps (NETs).^61^, ^62^, ^63^, ^64^ Furthermore, in murine abdominal sepsis, NETs recruit pro‐coagulant neutrophil MPs.^65^ Altogether, our findings demonstrate that leucocyte‐derived MPs generated under inflammatory conditions act as true pro‐senescent mediators of endothelial and vascular dysfunction and strongly suggest their contribution as noxious effectors of leucocyte recruitment to the damaged endothelium during IR.

Early consequences of IR remain difficult to decipher in‐vivo. SMPs therefore appear suitable tools in alternate approaches to assess IRI‐driven cellular activation and for the monitoring of drugs targeting neutrophil and monocyte endothelial interactions, eventually triggered by NETosis.^66^ Our spleen MP‐mediated endothelial senescence model is a reliable tool in deciphering mechanisms of ageing‐induced inflammatory responses as well as those of accelerated endothelial senescence during transplantation‐associated IR.^67^ Endothelial cyto‐protection of highly vascularized grafts such as pancreatic islets, and the control of pro‐senescent MP shedding might limit IR in the early stages of transplantation, preserves endothelium at sites prone to early vascular injury and delay its dysfunction.

## CONFLICT OF INTEREST

The authors of this manuscript have no conflict of interest to disclose.

## AUTHOR CONTRIBUTIONS

AE performed crosstalk experiments, analysed data and wrote the paper. RA performed ex vivo vascular response investigations; MA, AWQ and HE contributed to flow cytometry analysis; FZ measured microparticles, MK, GK, LA and SK contributed to the initial experimental design; VS discussed some aspects of the manuscript; LK supervised the experimental design and discussed the manuscript; and FT conceived and supervised the study, and wrote and revised the manuscript.

## Supporting information

Supplementary Material

## Data Availability

All data that support the findings of this study are available within the article and its supporting information file and from the corresponding author upon reasonable request.
