# Supplementary material for: Significance of neutrophil microparticles in ischaemia‐reperfusion: Pro‐inflammatory effectors of endothelial senescence and vascular dysfunction
Source: J Cell Mol Med. 2020 Jun 10;24(13):7266–81. doi: 10.1111/jcmm.15289 (PMC7339165; doi:10.1111/jcmm.15289)
Supplement: Supplementary file 1 — Supplementary Material [file JCMM-24-7266-s001.docx]

**Supporting information**

**Significance of neutrophil microparticles in ischemia-reperfusion: Pro-inflammatory effectors of endothelial senescence and vascular dysfunction**

Ali El Habhab^1^, Raed Altamimy^1^, Malak Abbas^2^, Mohamad Kassem^1^, Lamia Amoura^1^, Abdul Wahid Qureshi^1^, Hanine El Itawi^1^, Guillaume Kreutter^1^, Sonia Khemais-Benkhiat^2^, Fatiha Zobairi^1,5^, Valérie B. Schini-Kerth^1,3^, Laurence Kessler^1,4,5†^ and Florence Toti^1,3†^*

**Supporting Materials and Methods**

**Isolation, quantification and characterization of splenocyte-derived MPs**

After 24h splenocyte culture, supernatants were collected under sterile conditions. Splenocytes and cell debris were discarded by centrifugation at 300g, 15min at room temperature. Supernatants were centrifuged twice at 14000g, 60 min, 4°C, and washed SMPs pelleted and concentrated in Hanks Balanced Salt Solution (HBSS, without phenol red, without Ca^2+^ and Mg^2+^, Lonza, Belgium) and stored at 4°C for a maximum of 1 month.

The size distribution analysis of SMPs was performed by Tunable Resistive Pulse Sensing technology (TRPS) using the Izon qNano system. with the NP400 nanopore and 210-350 nm calibration beads (Izon Science Ltd, New Zealand). SMPs were detected in short pulses of the current blockades. The size distribution of SMPs was determined using the Izon control suite 3.3 software.

Biotinylated monoclonal antibodies to the following various cell types were insolubilized onto streptavidin-coated microtitration plates prior incubation with SMPs: (1) anti-CD45 for leucocyte common antigen, (2) anti-CD3 for T lymphocyte population, (3) anti-CD4 for T helper cells, (4) anti-CD8b for T cytotoxic cells, (5) anti-CD161a for natural killer cells, (6) anti-CD25 for IL-2 receptor T cells and splenic dendritic cells, (7) anti-CD31, a dual probe for apoptotic endothelial cells and platelets stimulation, (8) anti-CD11b/c for monocyte/macrophage and granulocyte, (9) anti-CD11b for neutrophils. All characterization antibodies were purchased from BD Pharmingen, San Jose, USA.

**Isolation of Coronary Artery Primary Endothelial Cells**

Briefly, left circumflex coronary arteries were excised from fresh hearts, cleaned of adhesive conjunctive tissues and the remaining blood flushed with cold phosphate-buffered saline (PBS) without calcium. ECs were further isolated by filling the artery with MCDB131 medium (Life Technologies SAS, St Aubin, France) supplemented with 100 U/ml penicillin, 100 U/ml streptomycin, 250 mg/ml fungizone, and 1 mM L-glutamine (all from Lonza, St Quentin en Yvelines, France) and containing 1 mg/ml type I collagenase (Worthington, Serlabo Technologies, Entraigues sur la Sorgue, France) for 15 min at 37°C. ECs were collected in the effluent after circular massages of the arteries and a final medium flush. Collected ECs were centrifuged at 400 g after and pelleted cells suspended in complete MCDB131 medium supplemented with 15% fetal calf serum. ECs from three different arteries were seeded in a T25 flask and expanded at 37°C in a 5% CO2 humidified atmosphere. ECs adhering to the bottom of the flask were selected by removing the medium containing non-adherent cells after 5-6 h incubation in complete MCDB131 medium and further grown for 48-72 hours (passage 0).

**Measurement of apoptosis and Senescence-associated β-galactosidase activity**

Apoptosis was detected by flow cytometry using Annexin-V and propidium iodide double labeling. P1ECs were harvested by trypsin treatment, washed and incubated with fluorescent Annexin-5 (5 μg/ml ImmunoTools, Friesoythe, Germany) and 2.5 μg/ml propidium iodide (Miltenyi Biotec SAS, Paris, France) for 15 min, at room temperature in the dark. The degree of apoptosis was measured as the proportion of Annexin-a5^+^/PI^+^ double stained cells by flow cytometry. Fluorescence acquisition was performed by Flow Cytometry (Guava EasyCyte Plus, Millipore). A minimum of 2000 events were analyzed.

Senescence-associated β-galactosidase activity (SA-β-gal) was measured in ECs by flow cytometry (FACScan, Becton Dickinson, San Jose, USA) after labelling with the fluorogenic cell permeable substrate C12FDG (5-dodecanoylaminofluorescein Di-β-D-galactopyranoside, Invitrogen, Illkirch, France). Briefly, ECs were pretreated with 300 μM chloroquine for 1h before addition of 33 μM C12FDG. After 1h incubation, ECs were washed with ice-cold PBS, trypsinized and analyzed immediately. Data were acquired and analyzed using the CellQuest software (Becton Dickinson). Light scatter parameters were set to eliminate dead cells and debris. The green C12-fluorescein signal was measured and SA-β-gal activity estimated using the mean fluorescence intensity (MFI). Auto-fluorescence gains were determined in unlabeled cells and set at the first logarithmic decade. Typically, in P3 cell suspensions, a maximum of 90% cells were labeled with a shift of mean fluorescence was observed from 18-20 MFI in P1 ECs to 55-65 MFI in senescent ECs.

The proportion of ECs with senescence-associated-β-galactosidase activity (SA-β-gal) was measured in the endothelial monolayer using light microscopy and the Senescence Cell Histochemical Staining Kit (Sigma) according to the manufacturer's guidelines. SMP_LPS-_ or SMP_PMA/I-_treated and untreated P1 ECs were washed in HBSS and fixed with a 2% formaldehyde/0.2% glutaraldehyde solution in PBS. After 3 PBS washings, cells were incubated at pH 6 in the SA-β-gal staining solution containing the 5-bromo-4-chloro-3-indolyl-beta-d-galactopyranoside (X-gal) galactosidase chromogenic substrate. Blue staining reflecting SA-β-gal activity was continuously assessed from 2h to 12h post substrate addition at 37°C in a CO_2_-free atmosphere, using P3 senescent ECs as positive control and senescent cells counted.

**Measurement of cellular and mitochondrial oxidative stress**

P1ECs were seeded in 6-well plates (2.10^5^ cells/well) 6h before treatment by SMP_LPS_ or SMP_PMA/I_ or by 100 µM H_2_O_2_ as a positive control of oxidative stress-induced senescence. P1ECs were treated by 2.5 μM DiHyroEthidium, a redox-sensitive red fluorescent dye, for 30 min or by 5 μM MitoSOX^TM^ Red, a mitochondrial superoxide indicator, for 10 min at 37°C. After three warm PBS washings, P1ECs were harvested by scraping in MCDB131 media before flow cytometry assessment. Light scatter parameters were set to eliminate dead cells and subcellular debris. The red ethidium signal was measured and ROS accumulation was calculated as a fold increase by comparison with untreated P1ECs (P1=100%). Auto-fluorescence gains were determined in unlabeled cells and set at the first logarithmic decade.

**Western blot**

After treatment, cells were washed twice with PBS and then lysed in extraction buffer (20 mM Tris/HCl, 150 mM NaCl, 1 mM Na_3_VO_4_, 10 mM sodium pyrophosphate, 20 mM NaF, 0.01 mM okadaic acid, 1% Triton X-100 (Euromedex, Souffelweyershem, France), a tablet of complete protease inhibitor (Roche), pH 7.5). Total proteins (20 μg) were separated on 8% or 12% sodium dodecyl sulfate-polyacrylamide gels at 100V for 2h and transferred onto polyvinylidene di-fluoride membranes (GE healthcare, Vélizy-Villacoublay, France) at 100V for 2h. Aspecific binding sites were saturated by incubation of membranes for 1h with Tris-buffered saline solution (TBS, Euromedex) containing 3% bovine serum albumin (BSA) and 0.1 % Tween. Membranes were incubated overnight at 4°C with blocking solution containing 3 % BSA and primary antibodies directly against: rabbit polyclonal eNOS (1:1000 dilution; BD Biosciences, Le Pont de Claix, France), mouse monoclonal iNOS (1:500 dilution; BD Biosciences, Le Pont de Claix, France), rabbit polyclonal p53, mouse monoclonal gp91^phox^, rabbit polyclonal p22^phox^, rabbit polyclonal AT1, mouse monoclonal anti-PAR1 (1:1000 dilution; Santa Cruz Biotechnology, Dallas, TX), mouse monoclonal p21, mouse monoclonal p16 (1:500 dilution; Santa Cruz Biotechnology), rabbit polyclonal p47^phox^, rabbit polyclonal cleaved caspase3, mouse monoclonal COX-1 (1:1000 dilution; Cell Signaling Technology, Danvers, MA), mouse monoclonal COX-2 (diluted 1:500 dilution; BD Biosciences), rabbit polyclonal ACE (1:1000 dilution; Abbiotec), TF (1:1000 dilution, Sekisui Diagnostics, Sekisui Virotech GbmH, Rüsselsheim, Germany), rabbit polyclonal anti-Akt, rabbit polyclonal anti-p38, rabbit polyclonal anti-ERK1/2, mouse monoclonal anti-JNK (1:1000 dilution, Cell Signaling Technology, US), mouse monoclonal anti-ICAM1, rabbit monoclonal anti-VCAM1 (1:1000 dilution, Abcam, UK), Anti β-tubulin (1:20000 dilution, abcam, UK). After washing, membranes were incubated with the peroxidase-conjugated corresponding secondary antibodies at RT for 1h (1:5000 dilution of anti- rabbit and anti-mouse immunoglobulin G; Cell Signaling Technology). Pre-stained markers (Invitrogen, France) were used for molecular mass determination. Immunoreactive bands were detected by enhanced chemiluminescence and their density analyzed using the ImageQuant acquisition system and analysis software (LAS4000 and ImageQuant TL 8.1, Amersham, UK). Data are expressed as a ratio to the housekeeping protein β-tubulin.

**Immunofluorescence studies**

To characterize the mechanism underlying the SMP-induced endothelial dysfunction, the expression level of several proteins involved in redox and inflammation responses were determined by immunofluorescence in frozen arteries, previously embedded in Tissue-Tek OCT (Sakura 4583, Leiden, The Netherlands), and cryosectioned at 14 μm. Sections were air dried for 15 min and stored at −80 °C until use. Sections were first fixed with 4% paraformaldehyde (Electron Microscopy Sciences, Hatfield, PA, USA), washed and treated with 10% milk in phosphate-buffered saline containing 0.1% Triton X-100 for 1h at room temperature to block nonspecific binding. Coronary artery sections were then incubated overnight at 4°C with antibodies directed against either eNOS (1/1000, 610297, BD Transduction Laboratories, Le Pont de Claix, France), (VCAM1 (1:1000, 134047, Abcam, UK) and COX-2 (1/250, 15191, Abcam, UK). For negative controls, the primary antibody was omitted. After washing with phosphate-buffered saline (PBS), the fluorescent secondary antibody was added (1/400, Alexa 633-conjugated goat anti-rabbit or anti-mouse IgG, A-21070 and A-21050, Thermo Fisher, Illkirch, France) for 2h at room temperature in the dark before PBS washing and mounted in Dako fluorescence mounting medium (Dako S3023, Les Ulis, France) and cover-slipped sections were evaluated by confocal microscopy using a confocal laser-scanning microscope (Leica TSC SPE, Mannheim, Germany). Quantification of fluorescence levels was performed using Image J software (version 1.49p for Windows, US National Institutes of Health).

**Supporting Tables**

**Table S1. Concentration of AV^+^-SMPs (Annexin V), CD45^+^-SMPs, CD3^+^-SMPs, CD4^+^-SMPs, CD8b^+^-SMPs, CD25^+^-SMPs, CD161a^+^-SMPs, CD31^+^-SMPs, CD11b^+^-SMPs, CD11b/c^+^-SMPs generated by splenocytes after 24h LPS or PMA/calcium ionophore stimulation**. Data are expressed as nM PhtdSer and mean ± SEM of experiments performed at least on three different cells cultures. Data are represented in figure 7 of the manuscript.

**
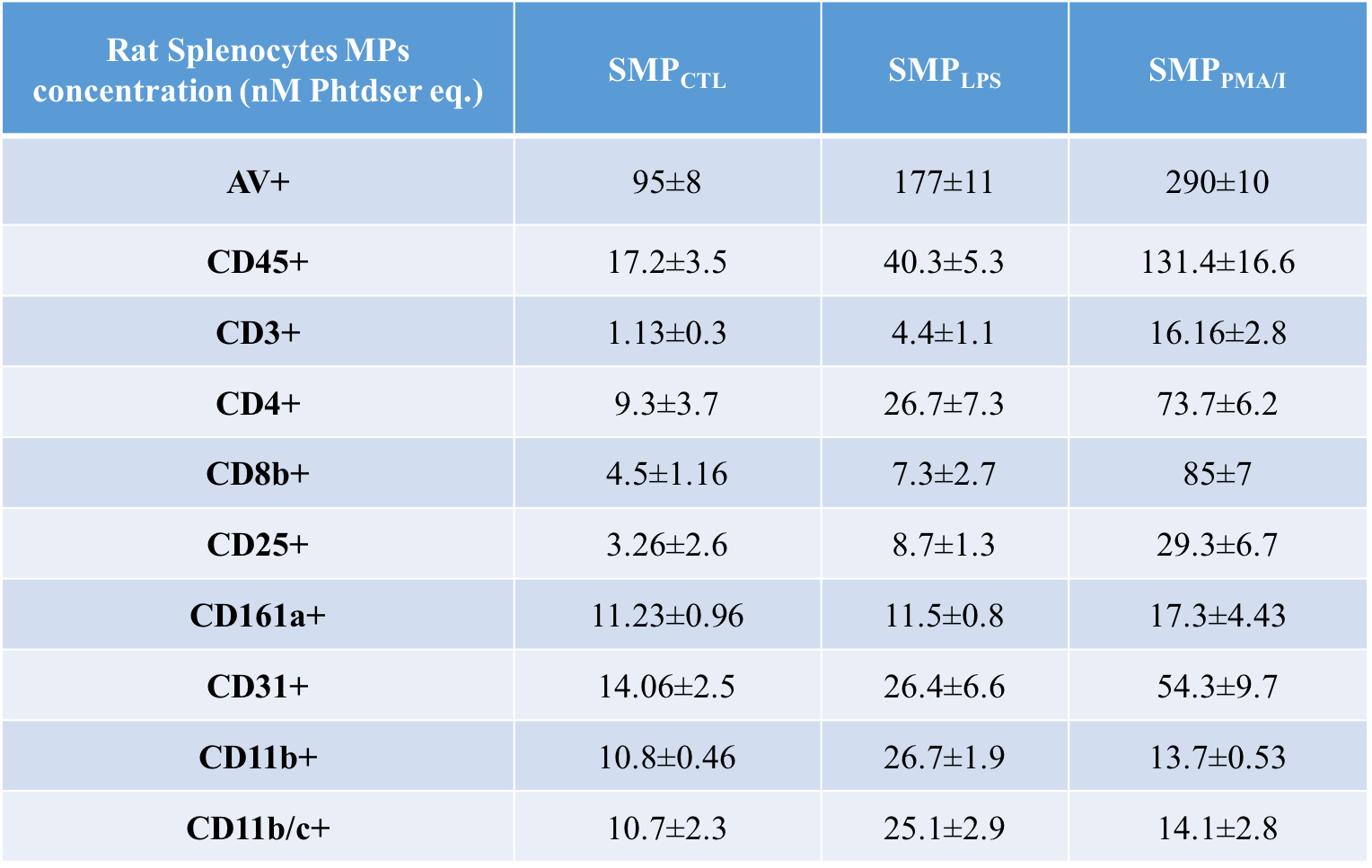
**

**Table S2. SMP concentration and characterization of their cell origin in total and immuno-depleted suspensions**. AV^+^-SMP_LPS_ (Total SMP_LPS_), CD31^+^-SMP, CD11b^+^-SMP, CD11b/c^+^-SMP were measured in concentrated and washed SMP suspensions obtained by 24h LPS treatment and after specific removal by immuno-depletion using magnetic beads coated with antibodies directed against CD31, CD11b, or CD11b/c.


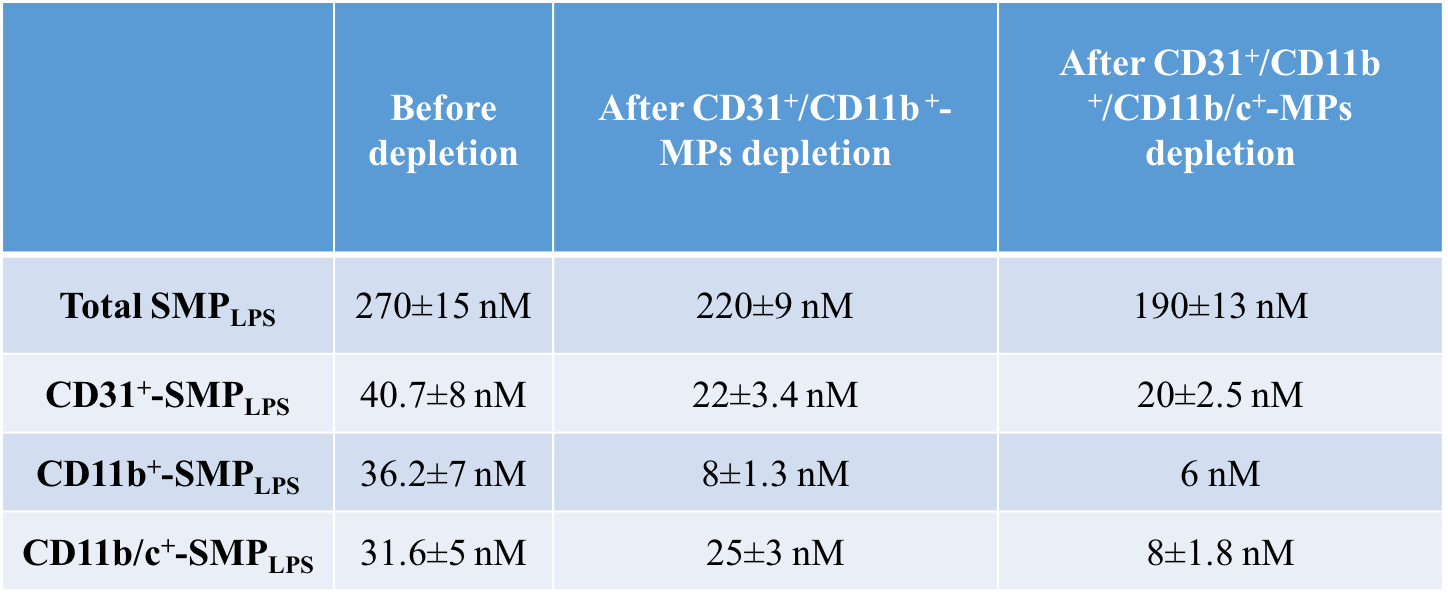


**Supporting Figures**

**
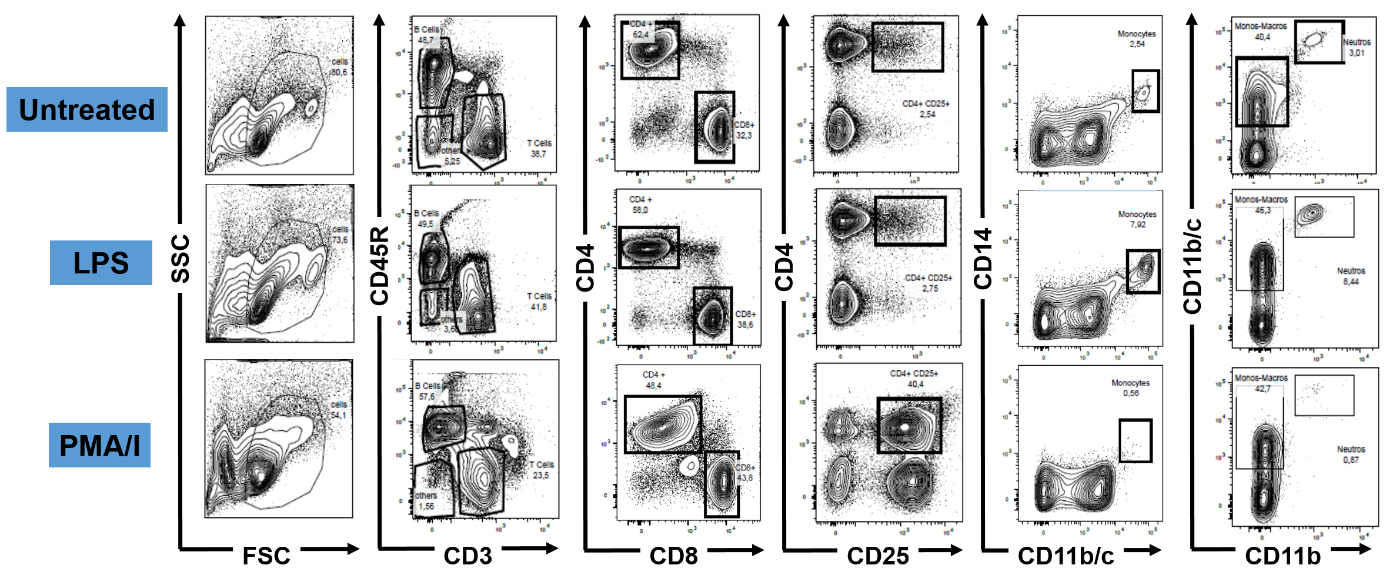
**

**Figure S1. Flow cytometry analysis of leukocyte subsets in the rat spleen.**

After 24h stimulation by either 5 µg/ml LPS or a combinaison of 25ng/ml PMA and 1 µM A23187 calcium ionophore, cultured splenocytes were washed and stained with antibodies to CD3 (pan T lymphocytes), CD4 (T4 lymphocytes), CD45R (B lymphocytes), CD11b/c (monocytes, macrophages and granulocytes), CD8 (T8 lymphocytes), CD25 (IL-2 receptor T lymphocytes), CD11b (neutrophils) and CD14 (monocytes). Flow cytometry analysis was performed to characterize the phenotype of each leukocyte sample. Representative dot plots showing the gating and the percentages of spleen leukocyte subsets in the indicated samples. *FSC: forward scatter; SSC: side scatter; Untreated: unstimulated splenocytes cultured for 24h*

**
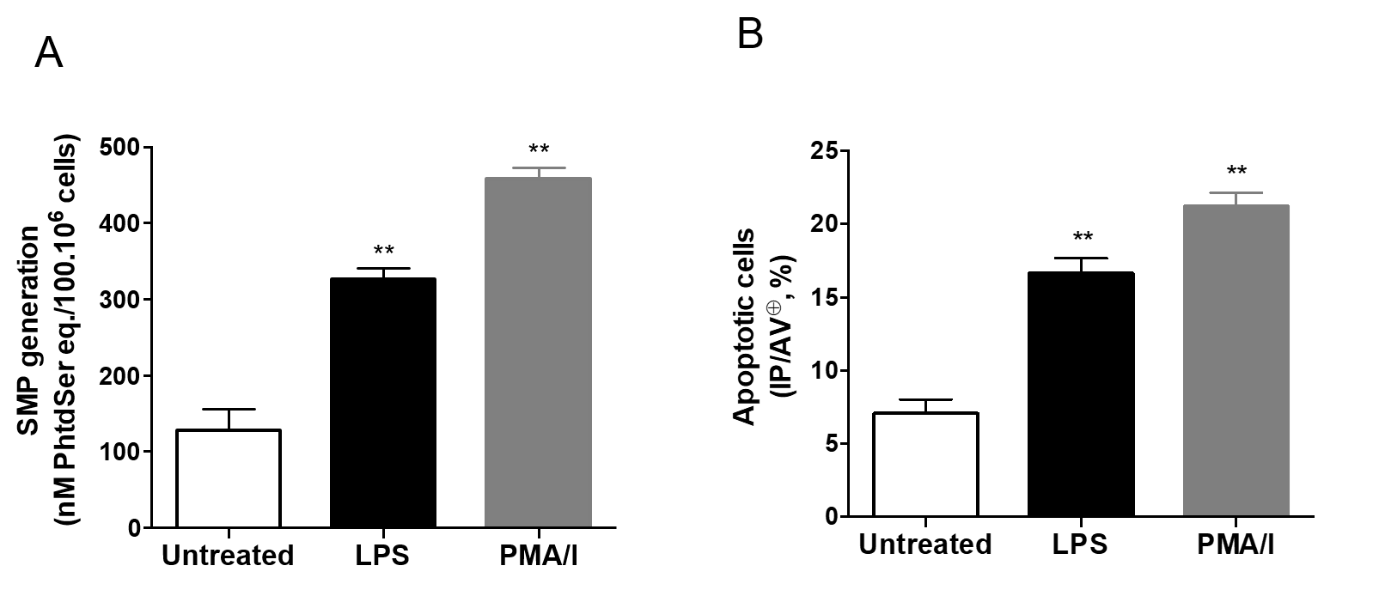
**

**Figure S2. Effect of LPS and PMA/calcium ionophore on the generation of microparticles by splenocytes and splenocyte apoptosis.**

Rat splenocytes (5.10^6^ cells/ml) in 20 ml were stimulated for 24h in the absence or presence of LPS or a combinaison PMA and calcium ionophore. (A) Rat splenocyte-derived micropaticles (SMP) in cell supernatant were measured by prothrombinase assay and expressed as nanomoles of phosphatidylserine equivalents (nM PhtdSer) per 10^8^ cells. (B) Splenocytes apoptosis was measured by flow cytometry using Annexin-V and propidium iodide double labeling.

*Untreated: Unstimulated rat splenocytes, LPS: Lipopolysaccaride, PMA/I: phorbol 12-myristate 13-acetate + A23187 calcium ionophore. Data are expressed as mean±SEM of experiments performed at least on three different cell cultures. ^**^P<0.01 vs untreated.*


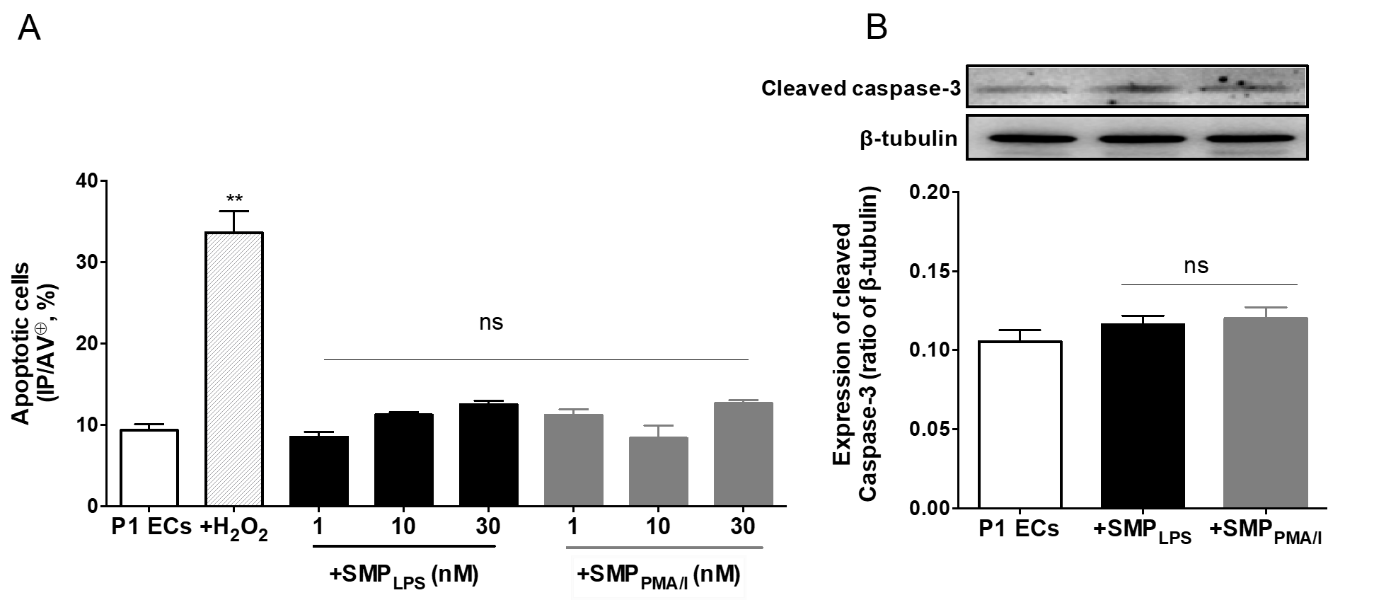


**Figure S3. Impact of SMPs from stimulated splenocytes on P1 young endothelial cells (ECs) apoptosis and cleaved caspase-3 expression.**

(A) P1ECs were incubated for 24h either with 100 µM H_2_O_2_, different concentrations of SMP_LPS_ or SMP_PMA/I_ (1-30 nM PhtdSer) before determination of the percentage of apoptotic cells using Annexin-5 and propidium iodide double labeling by flow cytometry. (B) P1ECs were incubated for 24h with SMP_LPS_ or SMP_PMA/I_ (30 nM PhtdSer) before determination of the expression of cleaved caspase-3 by western blot. Immunoblots (upper panel) and densitometry analysis of cumulative data (lower panel). Data are expressed as mean ± SEM of experiments performed at least on three different cell cultures. *ns: no significant vs P1 ECs, ^**^P<0.01 vs P1 ECs.*


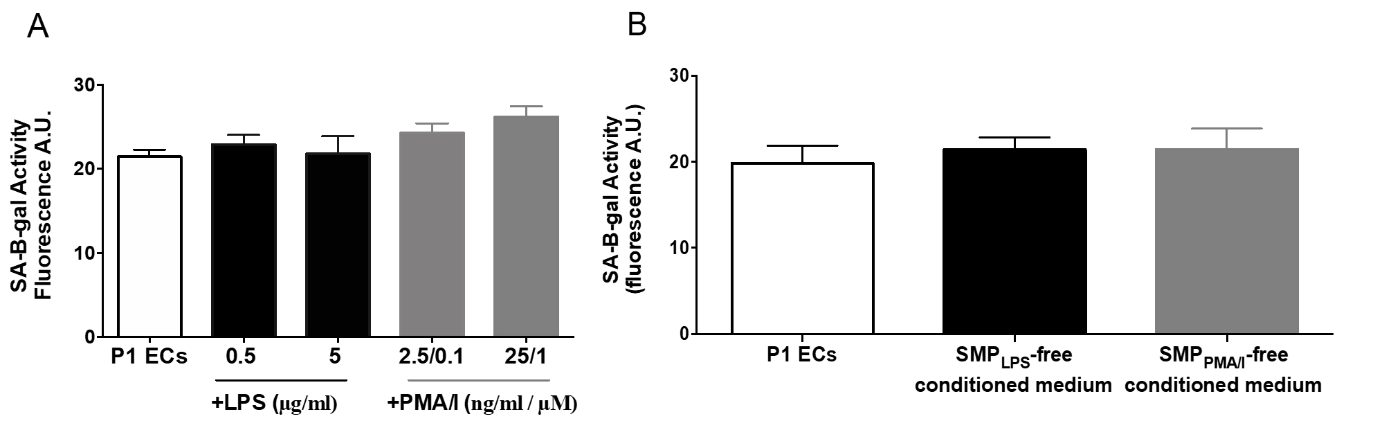


**Figure S4. LPS, PMA/I or truly soluble mediators from the supernatant of LPS- or PMA/I-treated splenocyte are not pro-senescent.**

(A) P1ECs were incubated for 48h with different concentrations of LPS or PMA/I. SA-β-gal activity was measured by flow cytometry. (B) P1 ECs were incubated for 48h in the absence (P1ECs) or presence of the conditioned medium of LPS- or PMA/I-treated splenocyte after removal of SMPs by a double step high centrifugation. SA-β-gal activity was measured by flow cytometry. Data are expressed as mean ± SEM of experiments performed at least on three different cell cultures.


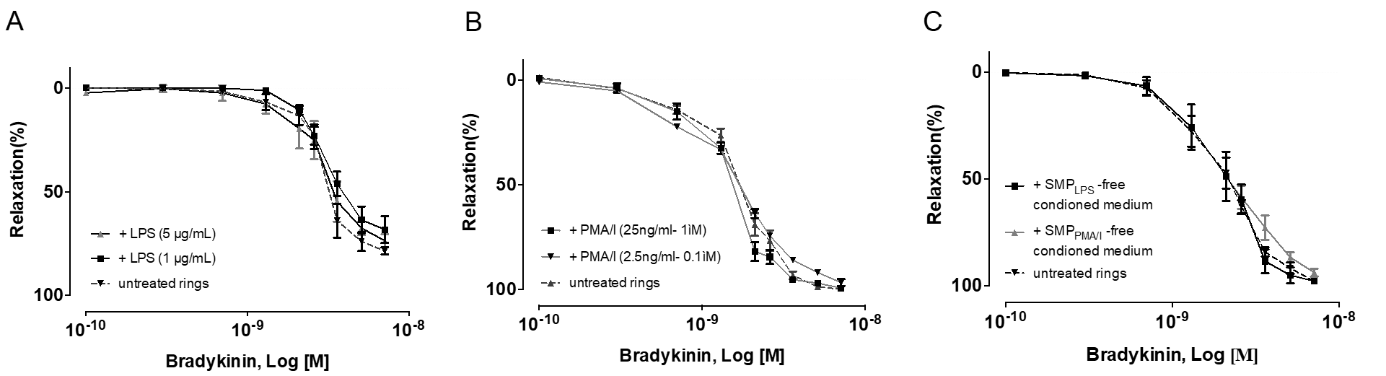


**Figure S5. Bradykinin endothelial-induced relaxation in coronary artery rings in the presence of LPS, PMA/I or truly soluble mediators from the supernatant of LPS- or PMA/I-treated splenocytes.**

Coronary artery rings were incubated for 12h with different concentrations of (A) LPS or (B) PMA/I or (C) in the presence of the conditioned medium of LPS- or PMA/I-treated splenocyte after removal of SMPs, before construction of concentration-response curves to bradykinin. Data are expressed as mean ± SEM of experiments performed at least on three different rings.
